# Neurovascular coupling, functional connectivity, and cerebrovascular endothelial extracellular vesicles as biomarkers of mild cognitive impairment

**DOI:** 10.1002/alz.14072

**Published:** 2024-07-03

**Authors:** Cameron D. Owens, Camila Bonin Pinto, Peter Mukli, Rafal Gulej, Faddi Saleh Velez, Sam Detwiler, Lauren Olay, Jordan R. Hoffmeister, Zsofia Szarvas, Mihaly Muranyi, Anna Peterfi, Ana Clara da C. Pinaffi‐Langley, Cheryl Adams, Jason Sharps, Zalan Kaposzta, Calin I. Prodan, Angelia C. Kirkpatrick, Stefano Tarantini, Anna Csiszar, Zoltan Ungvari, Ann L. Olson, Guangpu Li, Priya Balasubramanian, Veronica Galvan, Andrew Bauer, Zachary A. Smith, Tarun W. Dasari, Shawn Whitehead, Manoj R. Medapti, Fanny M. Elahi, Aikaterini Thanou, Andriy Yabluchanskiy

**Affiliations:** ^1^ Oklahoma Center for Geroscience and Healthy Brain Aging University of Oklahoma Health Sciences Center Oklahoma City Oklahoma USA; ^2^ Vascular Cognitive Impairment and Neurodegeneration Program Department of Neurosurgery University of Oklahoma Health Sciences Center Oklahoma City Oklahoma USA; ^3^ Department of Physiology Faculty of Medicine Semmelweis University Budapest Hungary; ^4^ International Training Program in Geroscience Doctoral School of Basic and Translational Medicine/Department of Public Health Semmelweis University Budapest Hungary; ^5^ Department of Neurology University of Oklahoma Health Sciences Center Oklahoma City Oklahoma USA; ^6^ Neuropsychology Service Department of Psychiatry and Behavioral Sciences University of Oklahoma Health Sciences Center Oklahoma City Oklahoma USA; ^7^ Department of Nutritional Sciences College of Allied Health University of Oklahoma Health Sciences Center Oklahoma City Oklahoma USA; ^8^ Veterans Affairs Medical Center Oklahoma City Oklahoma USA; ^9^ Cardiovascular Disease Section Department of Medicine University of Oklahoma Health Sciences Center Oklahoma City Oklahoma USA; ^10^ Department of Health Promotion Sciences College of Public Health University of Oklahoma Health Sciences Center Oklahoma City Oklahoma USA; ^11^ Peggy and Charles Stephenson Cancer Center University of Oklahoma Health Sciences Center Oklahoma City Oklahoma USA; ^12^ Department of Biochemistry and Molecular Biology University of Oklahoma Health Sciences Center Oklahoma City Oklahoma USA; ^13^ Vulnerable Brain Laboratory Department of Anatomy & Cell Biology Schulich School of Medicine & Dentistry London ON Canada; ^14^ Icahn School of Medicine at Mount Sinai New York New York USA; ^15^ James J. Peters Department of Veterans Affairs Medical Center Bronx New York USA; ^16^ Oklahoma Medical Research Foundation Oklahoma City Oklahoma USA; ^17^ Department of Medicine University of Oklahoma Health Sciences Center Oklahoma City Oklahoma USA

**Keywords:** endothelium, extracellular vesicles, functional connectivity, mild cognitive impairment, neurovascular coupling

## Abstract

**INTRODUCTION:**

Mild cognitive impairment (MCI) is a prodromal stage of dementia. Understanding the mechanistic changes from healthy aging to MCI is critical for comprehending disease progression and enabling preventative intervention.

**METHODS:**

Patients with MCI and age‐matched controls (CN) were administered cognitive tasks during functional near‐infrared spectroscopy (fNIRS) recording, and changes in plasma levels of extracellular vesicles (EVs) were assessed using small‐particle flow cytometry.

**RESULTS:**

Neurovascular coupling (NVC) and functional connectivity (FC) were decreased in MCI compared to CN, prominently in the left‐dorsolateral prefrontal cortex (LDLPFC). We observed an increased ratio of cerebrovascular endothelial EVs (CEEVs) to total endothelial EVs in patients with MCI compared to CN, correlating with structural MRI small vessel ischemic damage in MCI. LDLPFC NVC, CEEV ratio, and LDLPFC FC had the highest feature importance in the random Forest group classification.

**DISCUSSION:**

NVC, CEEVs, and FC predict MCI diagnosis, indicating their potential as markers for MCI cerebrovascular pathology.

**Highlights:**

- Neurovascular coupling (NVC) is impaired in mild cognitive impairment (MCI).
- Functional connectivity (FC) compensation mechanism is lost in MCI.
- Cerebrovascular endothelial extracellular vesicles (CEEVs) are increased in MCI.
- CEEV load strongly associates with cerebral small vessel ischemic lesions in MCI.
- NVC, CEEVs, and FC predict MCI diagnosis over demographic and comorbidity factors.

## 1 BACKGROUND

Cognitive dysfunction is a global health concern, with dementia projected to affect 152 million people by 2050.^1^ The risk of dementia increases with advanced age,^1^ and age‐related cellular and molecular alterations underly a driving force in cognitive impairment and dementia development.^2^ Current treatments for Alzheimer's disease (AD) and related dementias focus on addressing symptoms, with limited options for slowing the progression of early‐stage dementia.^3^ Mild cognitive impairment (MCI) precedes dementia and affects up to 18% of the population worldwide.^4^ MCI represents the earliest symptomatic stage of pathological cognitive decline,^5^ primarily manifesting as mild executive function impairments.^6^ Nearly one‐fifth of older adults’ progress from MCI to dementia annually.^7^ This underscores the need to determine shifts in brain function from healthy aging to MCI to provide mechanistic targets for intervention.

Neurovascular coupling (NVC) is a mechanism that coordinates the communication between parenchymal and vascular cell types to regulate cerebral blood flow (CBF) changes in response to neuronal activity.^8^ Preclinical models highlight the importance of NVC in sustaining cognitive function.^9^ Human studies demonstrate a strong association between cognitive performance and NVC responses in healthy older adults.^10^ Decreased NVC responses have been shown throughout the cortical and subcortical regions in individuals with AD and related dementias.^11^ Despite the significance of NVC in regulating CBF in response to cognitive demands, its alterations in MCI are not fully elucidated. Functional connectivity (FC) captures the statistical relationships within and between neuronal networks,^12^ constituting a critical element in cognition regulation.^13^ Research has shed light on aberrant changes in FC during rest and its association with neurocognitive conditions.^14^ However, the strength of relationships and regional alterations in FC during cognitive tasks that differentiate MCI from healthy aging are understudied.

Decreased CBF may precede dementia symptoms and pathologic parenchymal changes (i.e., amyloid‐β accumulation, neurofibrillary tangles, atrophy).^15^ Cognitive impairment in patients with MCI is associated with decreased CBF, supporting hemodynamic alterations prior to the development of AD and related dementias.^16^ Increased burden of ischemic changes in subcortical white matter is an age‐related change that is exacerbated in patients with MCI.^17^ Ischemic lesions are represented by white matter hyperintensities (WMH) on T2 fluid‐attenuated inversion recovery (FLAIR) magnetic resonance imaging (MRI), with deep WMHs representing a marker for small vessel disease.^18^ Higher WMH loads have a relationship with decreased CBF and FC strength in areas of cognitive processing.^19^ This highlights the negative impact of perfusion deficits in small vessel cerebrovascular accidents on the mechanisms crucial for cognitive function.

Extracellular vesicles (EVs) have emerged as a mechanism of intercellular communication^20^ and a potential marker of active disease, particularly in cardiovascular and neurocognitive conditions.^21^, ^22^, ^23^, ^24^ It is recognized that the cerebrovascular endothelium plays a critical role in regulating transfer of substances across the blood‐brain barrier to maintain parenchymal homeostasis and preserve cognitive health^25^ ; however, the roles played by cerebrovascular endothelial EVs (CEEV) in neurocognitive pathology is yet to be uncovered.

To fully understand the relationship between NVC and FC in individuals with MCI, we employed functional near‐infrared spectroscopy (fNIRS) measurements during the *n*‐back working memory paradigm. Blood was obtained from participants to determine the association of plasma CEEV levels with cerebrovascular small vessel ischemic burden. We hypothesized that impaired NVC responses and decreased FC would classify MCI with high feature importance and that CEEV levels would be elevated in MCI compared to CN. Integration of these physiological concepts will yield significant scientific advancements by (1) uncovering characteristic and functional determinants of MCI, thus bridging the gap for a lack of MCI mechanistic study; (2) improving predictive capability of neurocognitive disorders, leading to proactive clinical care in our rapidly aging population; and (3) providing translationally relevant results that will spur longitudinal clinical trials aimed at preventing cognitive impairment.

## 2 METHODS

### 2.1 Sex as a biological variable

Participants were matched based on age, and upon availability, by sex. Both male and female sexes were included in the study. Sex was included as a biological variable in random Forest to determine the importance of this variable in group classification of MCI.

**RESEARCH IN CONTEXT**

1. **Systematic review**: Cognitive task‐activated neurovascular coupling (NVC) and functional connectivity (FC) in mild cognitive impairment (MCI) have mixed findings. Cerebrovascular endothelial extracellular vesicle (CEEV) concentration has not been measured in MCI.
2. **Interpretation**: Aging is associated with impaired NVC and increased FC that acts as a compensatory mechanism preventing further cognitive decline. We show that in MCI, NVC is further decreased, and FC compensation is lost. CEEVs were increased in MCI and significantly associated with small vessel ischemic damage. NVC, CEEVs, and FC predicted MCI diagnosis with high accuracy. These findings contribute to the mechanistic determinants of cognitive impairment development.
3. **Future directions**: This manuscript determined pathophysiological changes in MCI, a potentially new biomarker for cerebrovascular pathology, and a new mechanistic avenue for clinical trials to mitigate the conversion from healthy aging to MCI. Alterations in molecular cargo of CEEVs in MCI may uncover pathways for how increased CEEVs may potentiate cerebrovascular damage.

### 2.2 Study participants

Community‐dwelling older adults and participants with MCI were recruited in this cross‐sectional observational study at the University of Oklahoma Health Sciences Center (OUHSC). All screening and data collection were conducted in the Translational Geroscience Laboratory of the Center for Geroscience and Healthy Brain Aging at the OUHSC. Participant screening was performed to determine eligibility prior to participation. Inclusion criteria consisted of age > 50 years old, Clinical Dementia Rating (CDR) equal to 0.5 and/or Montreal Cognitive Assessment (MoCA) <26 and ≥19 for the MCI group. Older adults were included in the CN group if there was no clinical diagnosis of MCI or any cognitive impairment, and MoCA ≥ 26. Participants with chronic conditions (e.g., type 2 diabetes, hypercholesterolemia, hypertension, major depressive disorder) that were pharmacologically uncontrolled, current or prior cerebrovascular complications (e.g., large vessel ischemic stroke with chronic functional impairment), neurodegenerative diseases, and any medical conditions or functional impairments which, in the opinion of the research and clinical neurology team, would render participants unable to complete the study were excluded from the study. All participants except three were right‐handed. All participants refrained from consuming caffeine on the day of measurement. Participants were enrolled into the study after signing informed consent.

### 2.3 Blood pressure

Blood pressure was measured in accordance with the 2017 AHA/ACC Hypertension Guideline for Standard Measurement of Blood Pressure. An average of up to three blood pressure recordings were calculated for each participant and systolic, diastolic, and mean arterial pressure (MAP) were compared between groups (CN vs. MCI).

### 2.4 Quantification of deep WMH

T2‐weighted FLAIR 1.5 Tesla MRI scans were used for ordinal quantification of subcortical deep white matter lesions by Fazekas scale. An experienced board‐certified, fellowship trained vascular neurologist, blinded to functional neuroimaging and EV patient values, graded the MRI scans for all participants. The Fazekas scale ranges from 0 (absent) to 3 (large confluent areas) and is a quick, simple, and reliable measurement to determine the deep and periventricular WMH load and associations with functional and biological variables.^26^

### 2.5 Neuropsychological testing and working memory paradigm

Participants were administered neuropsychological testing from the NIH Toolbox Cognition Battery as described.^27^ Tests selected included all fluid cognitive measures (i.e., dimensional change card sort, Flanker Inhibitory Control and Attention, Picture Sequence Memory, List Sorting Working Memory, pattern comparison, and oral symbol digit test) and crystalized ability measure (picture vocabulary test). The cognitive *n*‐back working memory paradigm was administered to participants as described.^27^ In brief, participants were seated in front of a computer monitor and were instructed to click the left mouse button when the target appeared. For 0‐back, participants were instructed to click the left mouse button when they saw a W appear on the screen. Participants were instructed to click the left mouse button when they saw a repeat letter (S‐D‐A‐A) for 1‐back, and when every other letter is repeated (X‐A‐T‐A) for 2‐back. Duration of each *n*‐back block was 72 s.

Fully corrected T‐score, which compares the test taker score to the NIH toolbox nationally representative normative sample and adjusts for demographic variables (i.e., age, sex, race/ethnicity, educational attainment), was used for group comparison. Fully corrected T‐scores have a normative, demographic‐adjusted population mean of 50 and SD of 10. A composite fully adjusted scale score for fluid cognition comprises dimensional change card sort, Flanker Inhibitory Control and Attention, Picture Sequence Memory, List Sorting Working Memory, and pattern comparison and was used to compare groups overall fluid cognitive ability in multiple cognitive domains. In addition, raw score (number of correct responses) was compared between groups for oral symbol digit test. To determine processing speed for each *n‐*back block, we calculated the average RT of correct responses as described.^27^ *n‐*back performance was measured first by calculating the hit rate (_HIT_) and false‐alarm rate (_FA_) as described.^28^ In this study we used the accuracy index (*d’*) to compare performance between groups. This score is defined as: $d^{\prime }=z_{Hit}\;-z_{FA}$, where z denotes z‐transform.^28^

### 2.6 fNIRS measurement protocol

Continuous changes in cerebrocortical hemodynamic signals during *n*‐back working memory paradigm were measured by fNIRS as described.^27^ In brief, participants wore a 128 port head cap (Easycap GmbH, Woerthsee‐Etterschlag, Germany) with 16 sources and 16 photodetectors (NIRx Medical Technologies LLC, Glen Head, NY) that covered the frontal cortex according to the international 10‐20 system. Sources emit light in the NIR range, 760 and 850 nm, to detect relative changes in deoxy‐ (HbR) and oxy‐ (HbO) hemoglobin. Forty‐eight source‐detector pairs were separated by 3 cm to sufficiently penetrate the cerebral cortex (∼1.5 cm), and all fNIRS signals were recorded with a sampling frequency of 3.9 Hz. The fNIRS montage is shown in Supplementary Figure 1.

### 2.7 Preprocessing and analysis of fNIRS recordings

fNIRS preprocessing and analysis were implemented in Matlab 2023b (Mathworks, Natick, MA) using custom scripts written by the authors and executed in a pipeline employing the Brain AnalyzIR toolbox (commit 46c645d).^29^ Participants were excluded if more than 80% of channels covering the prefrontal cortex had poor contact between optodes and the scalp^30^ and if any LDLPFC channel had poor contact. Motion artifacts and physiological signal components that do not arise from brain activity were removed as described.^27^ The modified Beer‐Lambert Law was applied to preprocessed optical densities and converted to chromophore (HbO and HbR) concentrations.^31^

For NVC responses, chromophores were prewhitened to reduce serially correlated effects from physiological and motion artifacts, and slow drifts were removed by a discrete cosine transform‐based high‐pass filter (0.009 Hz). General Linear Model (GLM) analysis of hemodynamic responses assessed NVC at the subject level, generating subject‐specific regression coefficients (β) for each *n*‐back block, chromophore, and brain region.

For FC, prior to application of the modified Beer‐Lambert Law, a fifth‐order Butterworth filter (0.0045‐0.4 Hz) was applied to reduce motion and systemic physiological noise contamination, as described previously.^10^ Following conversion of optical densities to chromophores, correlated HbO‐HbR motion‐related fluctuations were removed by correlation‐based signal improvement. After this final preprocessing step, neural‐evoked HbO and related HbR signal were summed (total hemoglobin [HbT]), as described.^32^ Pearson‐coefficients (r) were quantified for HbT for the entire duration of each *n*‐back block (72 s) for all channel pairs. Spurious correlations were removed by assigning 0 to negative *r* values, thus reflecting genuine functional brain region connections with significant (*p <* 0.05), positive *r* values. Graph theoretical analysis was used to characterize functional brain networks by regarding each channel as a “node” and each connection as an “edge.” Binary and weighted parameters were normalized to the maximum strength or number of connections for each node, respectively, yielding a value between 0 and 1. Calculated FC metrics based on graph theoretical analysis include: connection strength for each node (*^w^D_i_*), average connection strength for the whole graph (global connection strength – 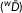), three channel (F3‐F5, F3‐F1, F3‐FC3) LDLPFC averaged connection strength 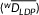, binary number of connections for each node (*D_i_*), average number of connections for the whole graph (global node degree – $\left(\bar{D}\right)$), and three channel (F3‐F5, F3‐F1, F3‐FC3) LDLPFC averaged number of connections $\left(\bar{D_{LDP}}\right)$. For further details, we refer the reader to our previous work for calculations of graph theoretical parameters.^10^

### 2.8 Blood draw and processing

Blood was collected from participants in two, 40 mL K2 EDTA Plus Blood Collection Tubes (BD Vacutainer). Blood was centrifuged at 2500 relative centrifugal force (rcf) for 15 min. Platelet‐poor plasma samples were stored at −80°C as 100 μL aliquots until the EV measurement. Freezing of samples was conducted for greater than 1 month. Previous studies indicate that extended periods of plasma sample storage for the purpose of EV extraction is an acceptable approach.^33^

### 2.9 EV measurement

Aliquots of plasma collected from participants were thawed and gently mixed. Ten microliters of plasma were added to 80 μL of filtered phosphate saline (PBS, 0.22 μm filtered). Each sample was labeled by adding 10 μL of antibody master mix containing antibodies labeling EVs of endothelial origin: CD31 (1:300, Alexa Fluor 488, cat#: ab215911, Abcam), CD144 (1:300, Alexa Fluor 488, cat#: 53‐1449‐42, eBioscience), CD105 (1:300, Alexa Fluor 488, cat#: 323210, BioLegend), lymphocyte origin (T cells): CD3 (1:300, Pacific Blue™, cat#: 300330, BioLegend), platelet origin: CD41 (1:300, Pacific Blue™, cat#: 303714, BioLegend), and for MAL positivity (1:100, Alexa Fluor 647, cat#: bs‐4835R‐A647, Bioss) in filtered PBS. Samples were mixed for 2 hours on ice, protected from light. Up to eight samples were labeled at the same time to reduce the effect of labeling time between the first and the last sample. For each stained sample, a nonlabeled sample was prepared by adding 10 μL of plasma to 90 μL of filtered PBS.

The differences in the concentration and ratios of EVs in plasma samples were measured using ApogeeFlow Micro‐Plus flow cytometer (ApogeeFlow, UK) (Supplementary Figure 2A). A solution containing silica and polystyrene beads ranging in size from 80 to 1300 nm (ApogeeMix, cat#: 1527, ApogeeFlow, UK) was used daily for the quality control of the instrument (Supplementary Figure 2B). Samples were measured at 1.5 μL/min flowrate for 120 s. Samples were diluted to the 10,000‐20,000 events/s concentration (100‐ to 400‐fold dilutions). For each subject, at least two different aliquots of plasma were measured during two separate measurement sessions.

Data were collected and analyzed using Histogram software (v6.0.117, ApogeeFlow, UK). The nonlabeled and fluorescence minus one (FMO) control samples were used to determine channel spillovers and gating strategy. To assess the plasma concentration of vesicles of cerebrovascular origin, the platelet (CD41^+^)‐ and lymphocyte (CD3^+^)‐negative events were gated out (Supplementary Figure 2C‐E). Subsequently, the concentration (events/μL) of double‐positive vesicles (endothelial‐ and MAL‐positive, CD31^+^/CD105^+^/CD144^+^ and MAL^+^) was determined for each measured aliquot. EVs with positive expression of pan‐endothelial markers, negative labeling of CD3 and CD41, and positive expression of MAL—specifically expressed in central nervous system (CNS) endothelial cells and not in peripheral endothelium^23^—distinguish CEEVs from EEVs. The ratios of cerebrovascular to vascular vesicles were assessed for each aliquot by dividing the number of endothelial‐ and MAL‐positive vesicles by the total number of vesicles of endothelial origin (endothelial^+^/MAL^+^ divided by endothelial^+^/MAL^+^ + endothelial^+^/MAL^−^) and expressed as percentage. Furthermore, overall CEEV concentrations were measured, averaged, and compared between groups. All concentration values were corrected by (multiplying by) dilution factor. For each subject, the average values from replicates were used. Representative images used in corresponding figures were created using FCS Express Pro 7 (v.7.18.0025, DeNovo Software).

### 2.10 Statistical analyses

Fisher's exact test, parametric and nonparametric two‐tailed unpaired t‐tests, two‐way analysis of variance (ANOVA), and Spearman's correlation between CEEVs and Fazekas scale were performed in Prism 10.0.2. We conducted a post hoc power analysis (G*Power 3.1) focused on the primary mechanism and our key area of interest—the NVC response in the left‐dorsolateral prefrontal cortex (LDLPFC). This was to confirm that our sample size was robust enough to lend credibility to the study's conclusions. Upon averaging the data from the LDLPFC across groups, with 19 participants in the CN group and 18 in the MCI group, we reached a statistical power of 0.8072. This indicates a strong likelihood that our study was adequately powered to detect a true effect. Educational attainment was separated by bachelor's degree and higher, and other, to not violate chi‐squared distribution assumptions. Brain AnalyzIR toolbox, implemented in MATLAB R2023b, was used for group‐level analysis of NVC responses. Correlation matrices, data preprocessing, feature selection, model training, and validation were performed in Python 3. All data presented were shown as mean ± SD unless otherwise stated. Parametric tests were only used if the Shapiro–Wilk test showed normal distribution. Nonparametric Mann–Whitney test was used to compare task‐averaged $\bar{D}$, task‐averaged LDLPFC NVC, CEEV%, CEEV concentration, EEV concentration, and MAL+ EV concentration. For two‐way ANOVA statistical test, which was used for all measures assessing the effect across *n‐*back tasks per group and size distribution of CEEVs, robust regression outlier removal (ROUT) method was used at the lowest tuning constant (*Q = *0.1%) to remove extreme outliers that would violate homoscedasticity and normality of residuals across groups in parametric statistical tests. Bonferroni post hoc tests were used for the assessment of group accuracy, RT and FC during the *n*‐back session, and CEEV size distribution. Spearman's correlation was used to assess the relationship between CEEVs and functional neuroimaging data and cognition (Jamovi 2.4.12.0).

For NVC responses individual β weights for channel, subject and *n*‐back task were obtained from first‐level GLM. Mixed effects second‐level GLM, fitted according to the Wilkinson–Rogers formula (‘β ∼ −1 + Group: session + (1|Subject)’, were evaluated by *t‐*contrasts for each channel to compare CN and participants with MCI (unpaired two‐tailed t‐test), and between condition (e.g., 1b vs. 0b_1) within group (paired t‐test). Type 1 error (from multiple [channel‐wise] comparisons) was mitigated by considering NVC responses as significant at *q <* 0.05 after controlling the FDR by Benjamini–Hochberg–procedure. Therefore, the pipeline was able to determine group‐related differences in NVC responses between groups, as well as determine the effect of task difficulty within group.

For prediction modeling, we determined an optimal classification model using a systematic approach that was adopted for data preprocessing, feature selection, model training, and validation. Given the relatively small dataset (*n *= 34) and the desire for a comprehensive evaluation, the process involved leave‐one‐out cross‐validation (LOOCV) to identify the best combination of features and model. In the process of building the classification model, a comprehensive data preprocessing and feature transformation strategy was employed. To standardize the scale of numerical features and facilitate convergence during the modeling process, using the StandardScaler, we standardized features by removing the mean and scaling to unit variance.  For the categorical variables, a one‐hot encoding scheme was implemented using OneHotEncoder. This encoding is needed for feeding categorical data to many estimators for machine learning models. The algorithm of choice, random Forest, was selected based on its capability to handle mixed feature types and low‐dimensional data effectively. It is noteworthy that this data‐driven approach was complemented by a predefined constraint on the model's complexity, specifically limiting the maximum features to three (k = 3). After model selection, feature importance was evaluated, and the final model was further tested by different test‐train split runs (*n* = 100) to ensure a comprehensive assessment of its performance. The aggregated metrics on the test set demonstrated consistent and reliable performance. F1 score was calculated as the harmonic mean of recall and precision.

## 3 RESULTS

### 3.1 Study participants

Characteristics and baseline physiological data from participants with MCI (*n = *20, 71.2 ± 8.0 years of age) and age‐matched controls ([CN] *n = *20, 70.8 ± 6.6 years of age) are summarized in Table 1. A significantly higher proportion of participants with MCI had ongoing, medically controlled psychiatric disease (*n = *13) compared to CN (*n = *5, *p = *0.0248), which includes depression, anxiety, and post‐traumatic stress disorder. We evaluated the diagnostic date of psychiatric disease, specifically depression, as this made up the largest percentage of psychiatric disease in participants with MCI (85% of psychiatric disease cases). This distinction is critical, as there is a bidirectional relationship between MCI and depression.^34^ Six of 11 participants with MCI were diagnosed with depression prior to MCI diagnosis. There was no significant difference in proportion of depression in CN (*n* = 2) compared to depression prior to MCI diagnosis (*n* = 6, *p *= 0.2351). Further, we investigated etiology of MCI based on data obtained from medical records and these data are presented in Supplementary Table 1.

**TABLE 1:** Patient demographics, conditions, medications, and baseline physiology.

Characteristics	CN (*n* = 20)		MCI (*n* = 20)	
	*n*	%	*n*	%
Sex				
Male	5	12.5	10	25.0
Female	15	37.5	10	25.0
Race				
American Indian or Alaska Native	1	2.5	0	0.0
Asian	0	0.0	0	0.0
lack or African‐American	1	2.5	0	0.0
Native Hawaiian or Pacific Islander	0	0.0	0	0.0
White or Caucasian	18	45.0	19	47.5
Other	0	0	1	2.5
Ethnicity				
Hispanic/Latino	0	0.0	0	0.0
non‐Hispanic / non‐Latino	20	50.0	20	50.0
Highest level of education				
Doctorate degree	7	17.5	2	5.0
Master's degree	2	5.0	1	2.5
Bachelor's degree	8	20.0	9	22.5
Associate degree	2	5.0	3	7.5
Other, no college degree	1	2.5	5	12.5
Active diseases				
Hypertension	6	15.0	11	27.5
Dyslipidemia	6	15.0	9	22.5
Type 2 diabetes mellitus	2	5.0	5	12.5
Hypothyroidism	3	7.5	1	2.5
Psychiatric disease	5	12.5	13	32.5
Neuropathy	3	7.5	6	15.0
Diseases of bones, joints, and muscle	10	25.0	9	22.5
Other medical conditions	19	47.5	15	37.5
Current smoker	0	0.0	0	0.0
Medications				
AT1‐receptor blocker	1	2.5	2	5.0
ACE‐inhibitor	2	5.0	6	15.0
β‐blocker	2	5.0	7	17.5
Ca‐antagonist	3	7.5	6	15.0
Diuretic	4	10.0	3	7.5
Statin	5	12.5	7	17.5
Hormone replacement therapy	4	10.0	3	7.5
Thyroid supplement	3	7.5	1	2.5
Other prescribed drugs	17	42.5	20	50.0
Baseline data	Mean	SD.	Mean	SD.
Age (years)	70.8	6.6	71.2	8.0
Systolic blood pressure (mmHg)	138.4	18.5	137.8	14.0
Diastolic blood pressure (mmHg)	86.9	8.8	82.5	10.2
Mean arterial blood pressure (mmHg)	104.0	12.0	100.9	10.9
Heart rate (beats per minute)	70.2	9.9	64.0	11.3
Body mass index (kg/m^2^)	24.6	4.9	27.8	5.8
Fazekas scale (0‐3)	n/a	n/a	1.19	0.5

*Note*: Ongoing medical conditions were pharmacologically controlled. Other conditions: gastroesophageal reflux disease, irritable bowel syndrome, chronic obstructive pulmonary disorder, hepatitis, herpes simplex virus, sleep apnea, asthma, chronic pancreatitis, dizziness, migraine, trigeminal neuralgia, carpal tunnel. Psychiatric disease: depression, anxiety, post‐traumatic stress disorder. Other prescribed drugs: ezetimibe, acyclovir, tramadol, trazadone, bisphosphonates, celecoxib, metformin, chronic obstructive pulmonary disorder medication, omeprazole, apixaban, escitalopram, buspirone, antiviral hepatitis medication, clopidogrel, carbamazepine, bupropion, insulin, dapagliflozin, cyclobenzaprine, semaglutide, memantine, donepezil.

**FIGURE 1:**
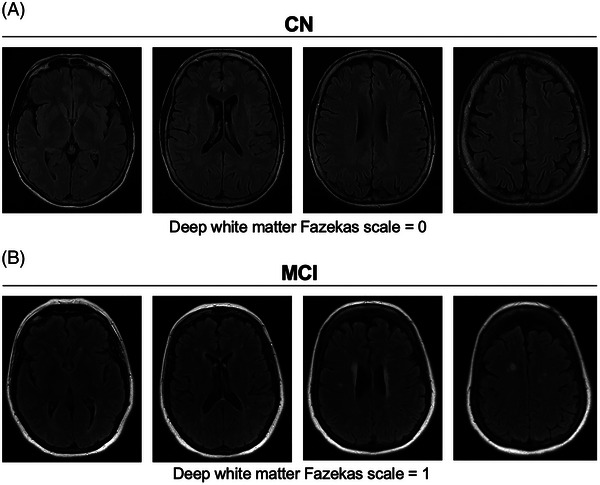
Clinical grading of small vessel ischemic lesions in control and participants with mild cognitive impairment (MCI). (A) Control (CN) participant (age: 68 years old, sex: female) with historical magnetic resonance imaging (MRI), recorded in 2023 shows a Fazekas scale grading of 0, indicating no small vessel ischemic damage determined by this grading classification. (B) Participant with MCI (age: 65 years old; sex: female) historical MRI, recorded in 2023, shows a Fazekas scale grading of 1, indicating moderate small vessel ischemic damage. Fazekas scale grading was assessed for deep white matter hyperintensities.

One CN participant had a historical T2 FLAIR MRI and 17 participants with MCI had historical MRI imaging. Participant MRIs were conducted within five years of participation in the study (median = 2022, IQR = 2021–2023). Figure 1 shows the representative image of one CN participant MRI (age: 68 years old, sex: female, Deep White Matter Fazekas scale = 0), and one participant with MCI (age: 65 years old, sex: female, Deep White Matter Fazekas scale = 1). All deep white matter Fazekas scale scores are shown in Supplementary Figure 3.

### 3.2 Fluid cognitive performance is diminished in participants with MCI while crystalized abilities are maintained in normal age range

**FIGURE 2:**
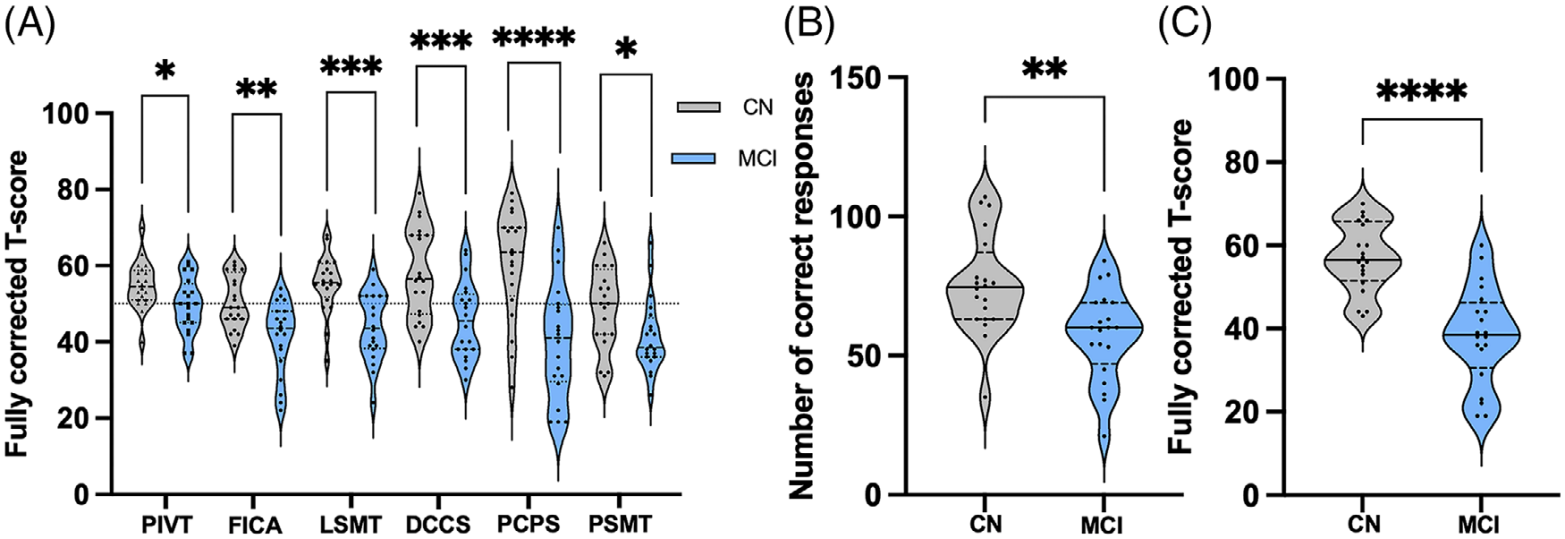
Fluid cognitive performance is diminished in participants with mild cognitive impairment (MCI), while crystalized intelligence is maintained in normal age rage. (A) Patients with MCI had a significant decrease in crystalized intelligence metric—picture vocabulary test (PIVT)—compared to control (CN), but maintained cognitive abilities in normal age, sex, race, and education range (Normative population mean = 50, SD = 10). Fluid cognition was impaired compared to age‐matched CNs in domains of inhibitory control and attention (FICA), working memory (LSMT), cognitive flexibility (DCCS), episodic memory (PSMT), and processing speed (PCPS). Fully corrected T‐score compares the test taker score to the nationally representative National Institutes of Health (NIH) toolbox sample, adjusting for age, sex, race, and educational attainment. (B) Processing speed was directly measured by oral symbol digit test and patients with MCI had decreased number of correct responses compared to age‐matched controls. (C) Fluid cognition composite scores, including performance on FICA, LSMT, DCCS, PCPS, and PSMT, indicate overall fluid cognitive deficit in MCI compared to age‐matched controls. Violin plots solid and dashed lines are presented as median and interquartile range, respectively; *n *= 20 participants per group. *p < 0.05, **p < 0.01, ***p < 0.001, ****p < 0.0001 by two‐tailed Student's *t*‐test (unpaired).

Cognitive decline in participants with MCI is confined to subdomains of fluid cognitive functioning (i.e., not dependent on past learned experience), with crystalized abilities significantly unaffected.^35^ To confirm these cognitive deficits in our cohort, we administered the NIH toolbox cognitive battery (Figure 2A‐C). Significant decreases in NIH toolbox fluid cognitive tasks, such as flanker inhibitory control and attention (CN: 50.40 ± 6.86, MCI: 41.30 ± 9.49, *p = *0.0013, [FICA]), list sorting working memory (CN: 55.25 ± 7.66, MCI: 44.00 ± 8.94, *p = *0.0001, [LSMT]), dimensional change card sort (CN: 58.35 ± 11.67, MCI: 45.65 ± 9.95, *p = *0.0007, [DCCS]), pattern comparison processing speed (CN: 60.80 ± 14.19, MCI: 40.15 ± 15.14, *p <* 0.0001, [PCPS]), picture sequence memory test (CN: 49.05 ± 9.633; MCI: 41.50 ± 10.51; p = 0.0230), and decreased number of correct responses in a pure processing speed task (CN: 75.45 ± 17.98, MCI: 57.50 ± 16.11, *p = *0.0020) were seen in participants with MCI compared to CN. There was a significant difference between CN and MCI participants in crystalized intelligence (CN: 54.85 ± 6.277; MCI: 49.70 ± 7.131; *p *= 0.202, Figure 2B); however, MCI participants were still within normal age, sex, education, and race range of crystalized intelligence (mean = 50, SD = 10). Fluid cognition composite score, which includes fluid measures FICA, LSMT, DCCS, PCPS, and picture sequence memory (PSMT), was decreased in participants with MCI compared to CN (CN: 57.10 ± 8.271, MCI: 38.45 ± 11.82, *p <* 0.0001) (Figure 2C).

**FIGURE 3:**
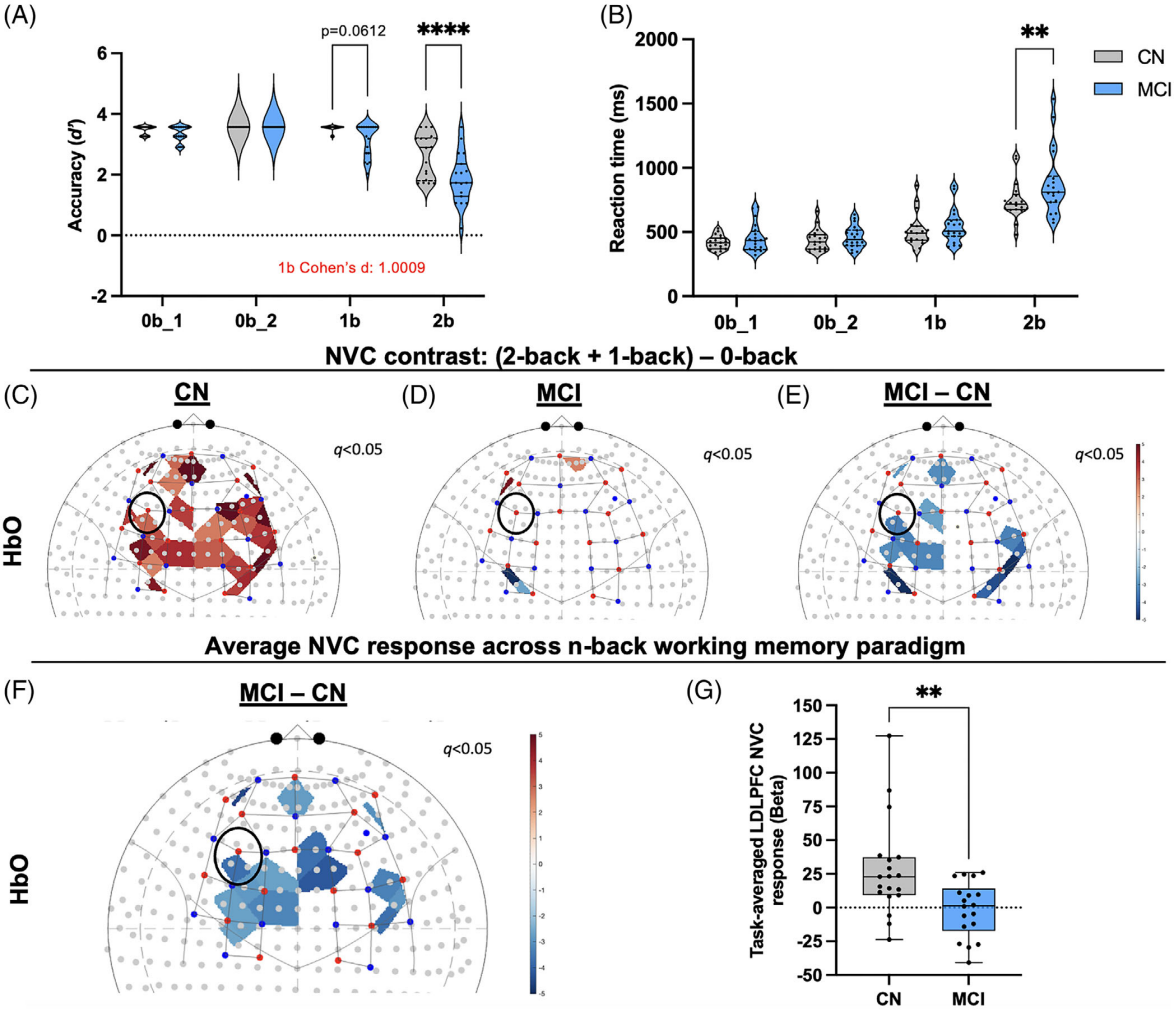
Working memory performance and neurovascular coupling (NVC) is impaired in participants with mild cognitive impairment (MCI). (A) Accuracy (d’) (see formula in main text) during 2b working memory *n*‐back task was decreased in MCI compared to age‐matched controls (CN) and (B) reaction time was increased during the most cognitively challenging 2b task. Panels C and D show the NVC response (relative change in oxy‐hemoglobin [HbO]) during the *n*‐back task. To determine the NVC response to cognitively challenging tasks, the contrast: (2‐b+ 1‐b) – 0b_2 was used for CN, MCI, and (E) group comparison (MCI – CN). The black circle represents the left dorsolateral prefrontal cortex (LDLPFC), a region responsible for working memory. (C) CN group showed increased NVC (red shaded areas), and (D) MCI group showed no change to increased cognitive workload across the prefrontal cortex. (E) Group analysis of this contrast showed decreased NVC (blue‐shaded areas) in the LDLPFC and medial prefrontal cortex in MCI compared to age‐matched controls. (F) Averaged NVC responses across the entirety of the *n*‐back paradigm determined that MCI patients had decreased NVC responses localized to the LDLPFC and medial PFC compared to controls. (G) Regression coefficients (beta) from (F) were extracted from the LDLPFC channels and show decreased NVC in MCI compared to age‐matched controls. Violin plots (A, B) solid and dashed lines are presented as median and interquartile range and box and whiskers graph (G) is presented as median (solid line), interquartile range (IQR) and minimum and maximum. Panels C, D, E, and F t‐contrast maps were generated using Brain AnalyzIR toolbox implemented pipeline based on General Linear Model approach. For further details see main text. For Panel A: 0b_1 *n *= 19 CN, 20 MCI, 0b_2 *n *= 15 CN, 16 MCI, 1b *n *= 15 CN, 19 CN, 2b *n *= 18 CN, 19 MCI. For Panel B: 0b_1 *n* = 19 CN, 20 MCI, 0b_2 *n* = 19 CN, 20 MCI, 1b *n* = 19 CN, 19 MCI, 2b *n *= 18 CN, 19 MCI. Panels A and B, robust regression outlier removal (ROUT) method was used for extreme outlier detection (tuning constant *Q *= 0.1%). **p < 0.01, ****p < 0.0001 by two‐way analysis of variance (ANOVA) with Bonferroni's multiple comparisons test. For Panels C–G: *n* = 19 CN, 18 MCI. Panels C–F: Colors refer to t‐statistics thresholded by *q *< 0.05 (obtained after false discovery rate [FDR] correction) Panel G: **p < 0.01 by unpaired t‐test. 0b_1, first 0‐back; 0b_2, second 0‐back; 1b, 1‐back; 2b, 2‐back; NVC, neurovascular coupling.

In addition to the NIH toolbox cognitive battery, we administered the *n‐*back working memory paradigm (Figure 3A‐B). Figure 3A shows the accuracy (*d’*) during each cognitive task and Figure 3B shows changes in reaction time (RT). One participant was removed from *d’* and RT 1‐back (1b) and 2‐back (2b) analysis in the MCI group and one participant was removed from 2b analysis in the CN group because they did not understand the task. An additional participant was removed from the CN group for all tasks because they clicked the wrong mouse button during the entire *n*‐back task. There was a significant decrease in 2b performance (CN: *n *= 18, MCI: *n *= 19, 95% confidence interval (CI) of predicted mean difference = [0.4064 to 1.168], *p *< 0.0001) in MCI compared to CN. Additionally, 2b RT was increased in MCI (*n* = 19) compared to CN (*n* = 18, 95% CI of predicted mean difference = [−255.3 to −26.54], *p *= 0.0088), indicating impaired working memory performance and processing speed during more cognitively challenging tasks.

### 3.3 NVC is impaired during the working memory task in participants with MCI

To determine NVC responses elicited by the *n*‐back task, we assessed changes in oxy‐hemoglobin concentration (HbO) in the frontal cortex within and between groups (Figure 2C‐G). Two participants with MCI and one participant from CN were excluded from analysis because they did not understand one or more of the tasks. Statistical contrast ((2b + 1b) – 0‐back)) was used to determine the effect of the more cognitively challenging tasks compared to the baseline, low cognitive demand task. This contrast showed a significant increase in NVC in the LDLPFC in the CN group (*n = *19) (Figure 2C) and no change in the LDLPFC in the MCI group (*n *= 18) (Figure 2D). Group comparison of this contrast showed significantly decreased NVC in the LDLPFC in MCI compared to CN (Figure 2E). All statistical contrasts for within and group comparison are presented in Supplementary Figure 4, which shows consistently decreased NVC in MCI compared to CN across *n*‐back conditions. Figure 2F shows a significant decrease in the LDLPFC NVC response across the entire *n*‐back paradigm in MCI compared to controls. Regression coefficient (β) values extracted from general linear model analysis showed that MCI had significantly decreased averaged LDLPFC NVC (F3‐F5, F3‐F1, F3‐FC3) responses (*n = *18, median = 1.401, IQR = [−17.27 to 14.16]) compared to CN (*n = *19, median = 22.86, IQR = [9.180 to 37.30, *p = *0.0033]) (Figure 2G). These data indicate that the LDLPFC, a region largely responsible for working memory performance,^36^ has decreased NVC in MCI compared to CN at higher cognitive loads and throughout the *n*‐back paradigm.

### 3.4 LDLPFC functional connections are decreased in participants with MCI during the working memory task

**FIGURE 4:**
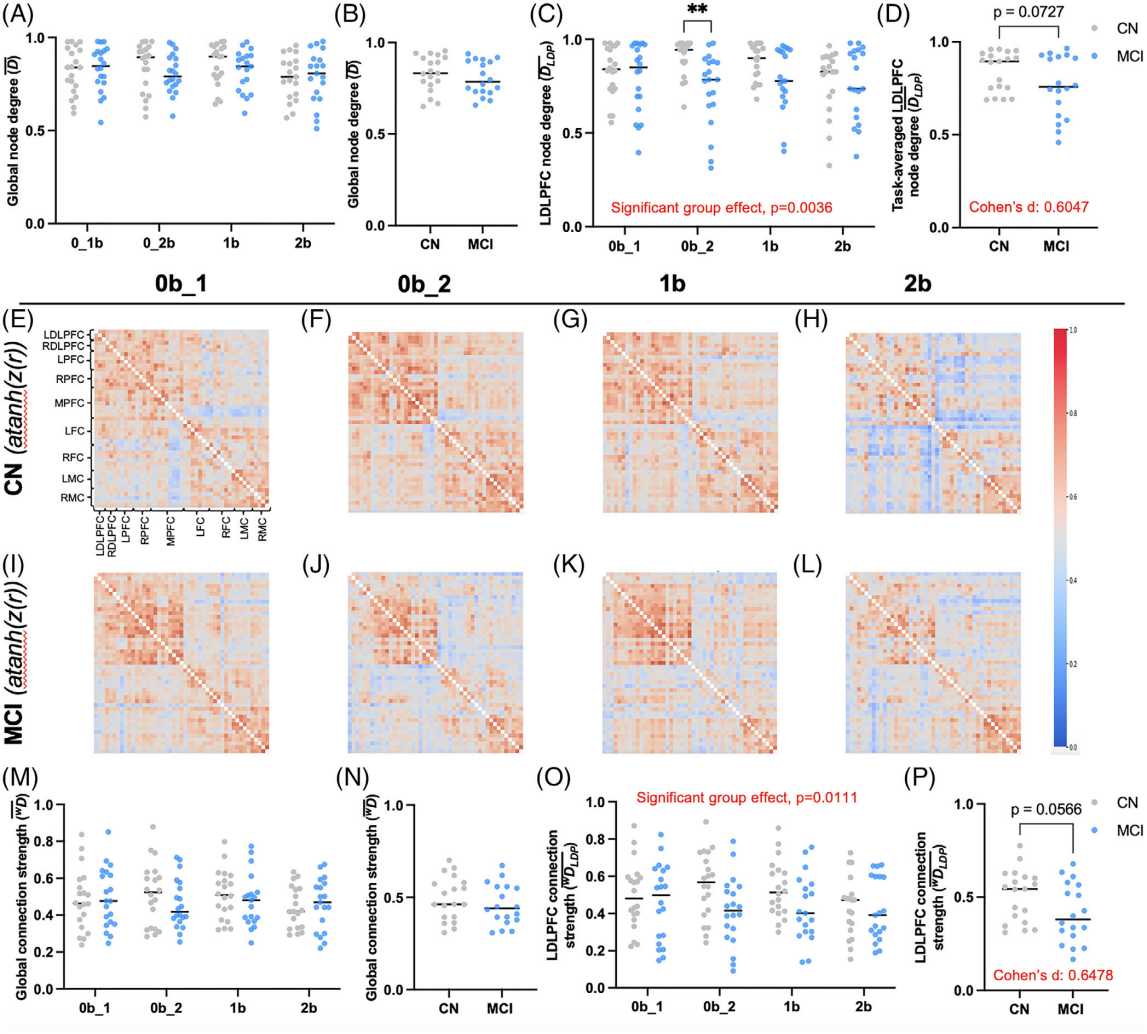
Functional connectivity (FC) is decreased in participants with mild cognitive impairment (MCI) during a working memory task. Individual global (i.e., referring to all functional near‐infrared spectroscopy (fNIRS) channels across the frontal cortex) network metrics show no change in number of normalized connections (i.e., node degree) in participants with MCI compared to age‐matched controls (CN) during each task (A) and all tasks averaged (B). Normalized left dorsolateral prefrontal cortex (LDLPFC) ‐averaged local node degree showed decreased number of connections in participants with MCI compared to CN irrespective of task (C) and all tasks averaged (D). Correlation matrices (E‐L) determine the strength of surrogate‐thresholded Pearson‐correlation (*p <* 0.05) coefficient values between each channel. Matrix connection values are inverse Fisher‐z transformed (i.e., atanh(z(r))) to obtain a normally distributed sample then averaged across the duration of each *n*‐back task (72 s). These data show increased connection strength (dark red) in and between areas of the LDLPFC in CN (E‐H) compared to participants with MCI (I‐L). Global connection strength, a global network metric representing the average number of connections adjusted relative to the maximum connection strength showed no change between MCI and CN (M) and *n*‐back task‐averaged connection strength (N). Following similar analytical measures as global connection strength (see main text for details), LDLPFC significant strength of connection to the rest of the cortex determined decreased LDLPFC connection strength irrespective of task (O), and a trend of decreased connection strength when all *n*‐back tasks were averaged in patients with MCI compared to CN (P). For Panels A, C, M, O: 0b_1 *n* = 20 CN, 20 MCI, 0b_2 *n* = 20 MCI, 1b *n* = 20 CN, 19 MCI, 2b *n *= 19 CN, 19 MCI following ROUT method; *p < 0.05, **p < 0.01, ***p < 0.001, ****p < 0.0001 by 2‐way ANOVA with Bonferroni's multiple comparisons test. For Panel C 0b_2 *n* = 18 CN and Panels A, M, O *n* = 20 CN following the ROUT method. For Panel B, D, N, P: *n* = 19 CN, 18 MCI; *p < 0.05, **p < 0.01 by Mann–Whitney unpaired *t‐*test.

To determine the number of functional connections during different levels of difficulty of working memory *n‐*back task, we calculated local network metrics for each fNIRS channel, local node degree $\bar{(D_{loc}}),$ and are presented in Supplementary Figure 5 as group averages. Group comparison of $\bar{D_{loc}}$ (Supplementary Figure 5A‐D) show decreased number of functional connections in the LDLPFC in participants with MCI compared to CN during the second 0‐back (0b_2) and 1b (*p *< 0.05). Subsequently, we calculated normalized global node degree $\bar{(D})$, a global network metric representing the average number of connections adjusted relative to the total possible connections. There was no difference in $\bar{D}$ between groups (Figure 4A). We averaged $\bar{D}$ for the entire *n*‐back paradigm, defined as task‐averaged $\bar{D}$, for each group. We determined that there was no difference in task‐averaged $\bar{D}$ between MCI and CN (Figure 4B).

We calculated normalized LDLPFC node degree $\bar{(D_{LDP}})$, representing the average number of functional connections between the LDLPFC and the rest of the cortical channels. There was decreased $\bar{D_{LDP}}$ in MCI compared to CN irrespective of cognitive task (95% CI of mean difference = [0.0254 to 0.1273], *p* = 0.0036), and a significant decrease in $\bar{D_{LDP}}$ during the 0b_2 task following multiple comparisons (CN: *n* = 18, MCI: *n* = 20, 95% CI of mean difference = [0.0326 to 0.2963], *p* = 0.0078) (Figure 4C). $\bar{D_{LDP}}$ was averaged for the entire *n*‐back paradigm, defined as task‐averaged $\bar{D_{LDP}}$, for each group. Task‐averaged $\bar{D_{LDP}}$ revealed a trend toward decreased FC in participants with MCI (*n* = 18, 0.7590 ± 0.1650) compared to CN (*n* = 19, 0.8429 ± 0.1062, Cohen's D = 0.6047, *p *= 0.0727) (Figure 4D).

### 3.5 LDLPFC strength of functional connections is decreased in participants with MCI during the working memory task

We determined local measures of strength of functional connections (surrogate‐thresholded Pearson‐correlation coefficient, $r_{ij}^{*}$) and weighted global and LDLPFC local node degree (*^w^* $\bar{D}$ *, ^w^* $\bar{D_{LDP}}$, respectively) in CN and participants with MCI. Each value in Figure 4E‐L correlation matrices shows the inverse Fisher‐transform $r_{ij}^{*}$ value obtained by the atanh function for channel‐to‐channel connection strength of all *n*‐back conditions. These data show higher connection strengths in CN localized to within and between regions of the LDLPFC compared to participants with MCI.

We then calculated *^w^* $\bar{D}$, a global network metric representing the strength of the average number of significant connections adjusted relative to the maximum connection strength. There was no difference between groups in *^w^* $\bar{D}\;$(Figure 3 M). *^w^* $\bar{D}$ were then averaged for the whole *n*‐back paradigm, defined as task‐averaged *^w^* $\bar{D}$, for each group. We determined no significant difference in task‐averaged *^w^* $\bar{D}$ in MCI compared to CN (Figure 4N).

Normalized LDLPFC weighted local node degree (*^w^* 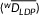 was calculated, representing the average strength of significant connections (*p *< 0.05) between the LDLPFC and the rest of the cortical channels. *^w^* 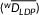 was decreased in MCI compared to CN irrespective of task condition (95% CI of mean difference = [0.0168 to0.1283], *p* = 0.0111) (Figure 4O). *^w^* 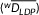 were then averaged for the entire *n*‐back paradigm, defined as task‐averaged *^w^* 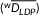, for each group. These data show a trend of decreased task‐averaged *^w^* 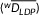 in MCI (n = 18, 0.4127 ± 0.1589) compared to CN (*n* = 19, 0.5095 ± 0.1393, *p *= 0.0566) (Figure 4P). To determine connection strength from each channel to all frontal cortical nodes, we calculated local network connection strength metrics, 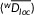 (Supplementary figure 5E‐H). These data show decreased LDLPFC connection strength in MCI compared to CN in 0b_2 and 1b (*p <* 0.05).

### 3.6 Participants with MCI have an increased load of CEEVs and are associated with high white matter hyperintensity levels

**FIGURE 5:**
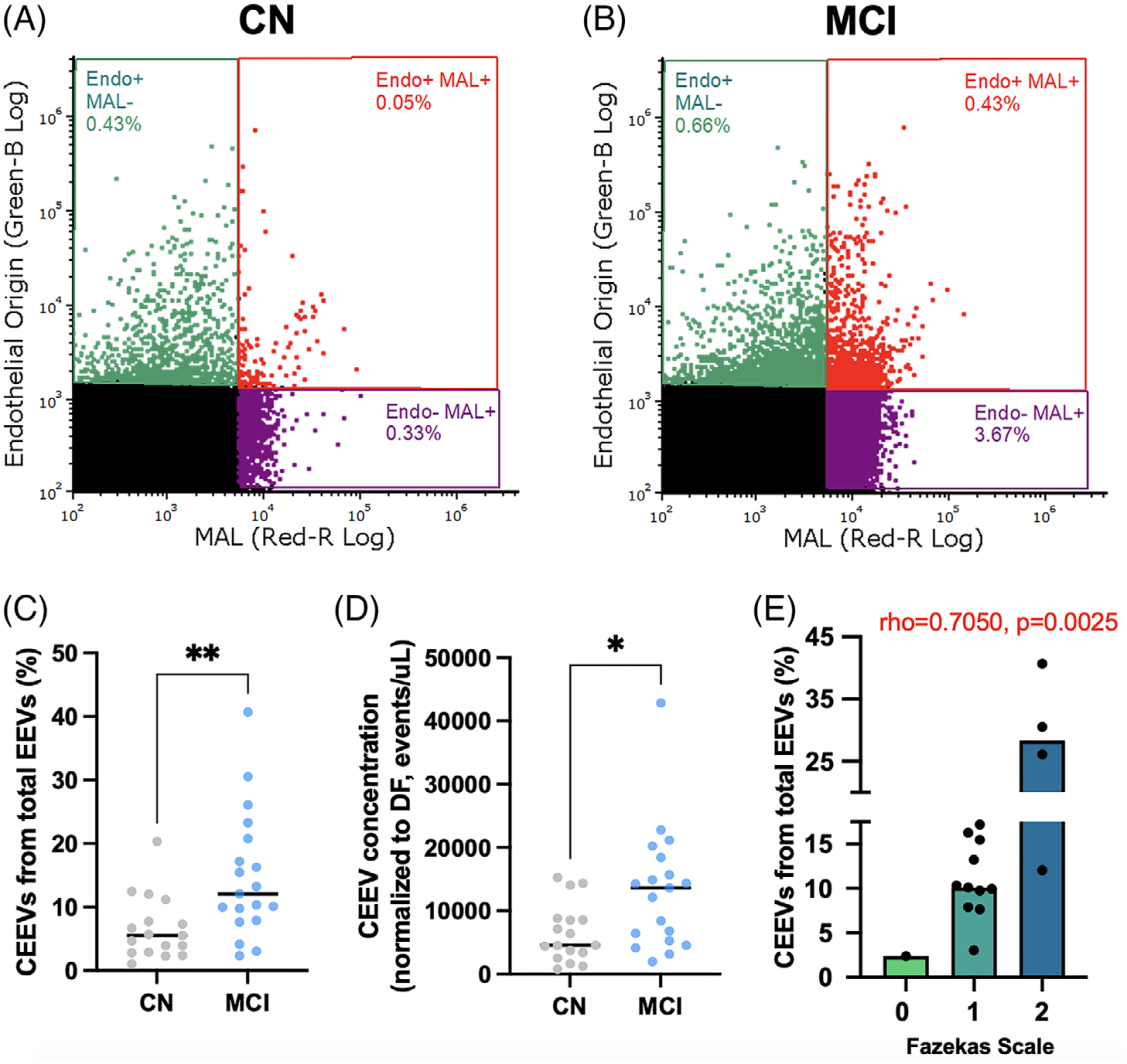
Participants with mild cognitive impairment (MCI) have increased cerebrovascular endothelial extracellular vesicles (CEEVs). Representative image of age‐matched control (CN) (A) and MCI (B) CEEV load. The ratios of cerebrovascular to other vascular vesicles were assessed by dividing the number of endothelial‐ and MAL‐positive vesicles by the number of all vesicles of endothelial origin (EEV) (endothelial+/MAL+ divided by endothelial+/MAL+ + endothelial+/MAL‐) and expressed as percentage (C). Panel C shows a significant increase in CEEV% in MCI compared to CN. Panel D shows a significant increase in CEEV concentration (events/μL) in MCI compared to CN. Panel E shows distribution of CEEVs to patients Fazekas scale grading. CEEV% had a significant correlation to increased WMH load. *p < 0.05, **p < 0.01 by Mann–Whitney unpaired *t‐*test. Panel E rho and *p* values were obtained by nonparametric Spearman correlation.

Ratio (%) and plasma concentration (events/μL) of CEEVs were measured in patients’ plasma (Figure 5A, B). We showed elevated ratio of CEEVs relative to total endothelial vesicles (EEVs) in MCI (*n *= 19, median = 12.05%, IQR = [7.906 to 20.78]) patient plasma compared to CN (*n *= 17, median = 5.535%, IQR = [2.849 to 9.455, *p *= 0.0024) (Figure 5C), and increased of CEEV concentration in MCI (*n *= 19, median = 13600 events/μL, IQR = [5260 to 18430) compared to CN (*n *= 17, median = 4545 events/μL, IQR = 2945 to 8675, *p *= 0.0147) (Figure 5D). There was no difference in concentration of EEVs between groups (CN: median = 98435 events/μL, IQR = [68625 to 126703], MCI: median = 92300 events/μL, IQR = [64460 to 118515], *p *= 0.6611) (Supplementary Figure 6A), suggesting that the observed change in the CEEVs numbers was not due to the change in the numbers of EEVs.

Current evidence indicates that the prevalence of EVs of larger sizes (>1000 nm) may be indicative of increased apoptotic mechanisms.^37^ To investigate whether there was an indication for an increase of proapoptotic pathways in the MCI group, we performed analysis of size distribution of CEEVs. There were no statistical differences in size distribution in CN versus MCI at less than 180 nm (predicted mean difference = −1309, *p *= 0.1238), 180‐600 nm (predicted mean difference = −848.2, *p *> 0.5960), 600‐1000 nm (predicted mean difference = −224.3, *p *> 0.9999), and in CEEVs of size greater than 1000 nm (predicted mean difference = −101.5, *p *> 0.9999) (Supplementary Figure 6B).

Myelin and lymphocyte (MAL) labeling was used to identify endothelial cells of cerebral origin.^23^ We showed no difference in other cell populations positive for MAL following negative gating of endothelial, lymphocyte and platelet EVs (CN: median = 51935 events/μL, IQR = [31420 to 190500], MCI: median = 109900 events/μL, IQR = [11615 to 476000], *p* = 0.5944) (Supplementary Figure 6C).

Correlations of CEEVs with functional neuroimaging and cognitive measures are shown in Supplementary Table 2. We determined that CEEVs had a significant negative correlation with *^w^* $\bar{D_{LDP}}$ and $\bar{D_{LDP}}$ (*n* = 34, Spearman's rho = −0.396, *p *= 0.021; Spearman's rho = −0.348, *p *= 0.044, respectively) and a significant negative relationship with fluid composite score (*n *= 34, Spearman's rho = −0.502, *p *= 0.002). CEEV ratio had a significant positive correlation with Fazekas scale grading in patients with MCI (*n *= 16, Spearman's rho = 0.7050, *p *= 0.0025) (Figure 5E). These exploratory analyses determined that CEEVs are associated with WMH burden in participants with MCI and that participants with MCI had a higher level of CEEVs than controls.

We investigated the effects of common hypertension medications (e.g., Ca^2+^ antagonists, angiotensin‐converting enzyme [ACE] inhibitors) and dyslipidemia medications (e.g., statins) and grouped patients by either hypertension medication or no hypertension medication and dyslipidemia medication and no dyslipidemia medication irrespective of MCI and CN status (Supplementary Figure 6D). There was no difference between either grouping, further substantiating CEEVs as a marker of cognition and cerebrovascular disease in MCI.

### 3.7 Diminished LDLPFC NVC responses, CEEVs, and FC predict MCI diagnosis

To determine if the impaired NVC responses, decreased LDLPFC FC, and increased ratio of CEEVs seen in our MCI cohort can predict group classification, we used feature selection process to identify the most informative variables for the final model. For the NVC variable (LDLPFC NVC), participant β values from LDLPFC channel‐averaged NVC response across the duration of the *n*‐back task were used. For FC, LDLPFC task‐averaged *^w^* $\bar{D_{LDP}}\;$ and $\bar{D_{LDP}}$ were assessed to determine group classification. Additionally, known factors that have shown predictive value to classify MCI, such as age, sex, hypertension,^7^ and depression prior to MCI diagnosis,^34^ were variables in our feature selection process. Sample size for our group classification analysis consisted of all patients who understood, and successfully completed all tasks of the *n‐*back task and had plasma samples available for CEEV analysis (CN *n *= 17, MCI *n = *17).

**FIGURE 6:**
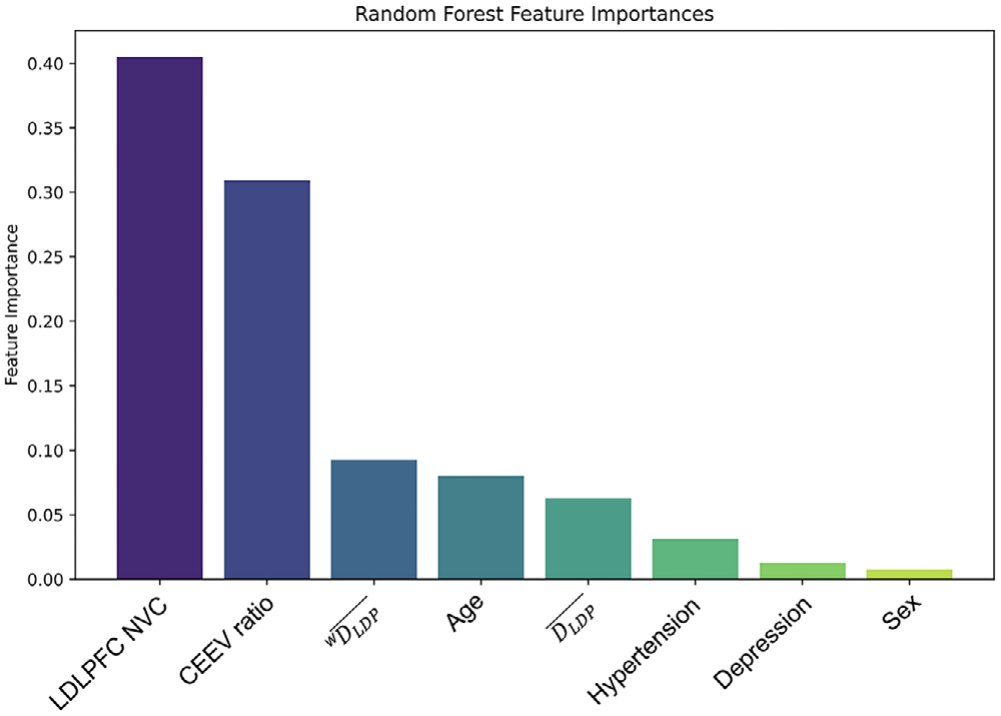
Neurovascular coupling (NVC), cerebrovascular endothelial extracellular vesicles (CEEVs), and functional connectivity (FC) are the most informative features in mild cognitive impairment (MCI) classification. We determined an optimal classification model using random Forest integrated with leave‐one‐out cross‐validation (LOOCV). Feature importance scores were assigned to variables, highlighting the model's predictive capability. Variables selected for analyses were neurovascular measures (NVC and FC metrics), CEEVs, and data either known to contribute to progression from aging to MCI, and/or data that had a trend toward a differential distribution between groups. The top three features were left dorsolateral prefrontal cortex (LDLPFC) NVC, CEEV ratio, and task‐averaged LDLPFC connection strength (*^w^*) with importance values of 0.4052, 0.3091 and 0.0926, respectively. These features were selected for the final model and following 100 runs with different test‐train splits, the chosen model showed a mean predictive accuracy of 68.00%, SD 16.00%. Individual model evaluation, highlighting its validity, achieved a precision of 88.57%, recall of 85.71% and an F1 score of 85.08%. *^w^* $\bar{D_{LDP}}$, weighted task‐averaged LDLPFC node degree; $\bar{D}$, task‐averaged global node degree; *^w^* $\bar{D}$, weighted task‐averaged global node degree.

Random Forest integrated with 34 runs of LOOCV assigned importance scores to each feature with LDLPFC NVC having the highest Gini feature importance value (0.4052) followed by CEEV ratio (0.3091) and *^w^* $\bar{D_{LDP}}$ (0.0926). These scores reflect the contribution of each feature to the model's predictive capability (Figure 6, Supplementary Figure 7). This meticulous methodology, rooted in a data‐driven approach with predefined constraints, contributes to the establishment of a reliable and interpretable classification model for small datasets. After feature selection, the three variables with the highest Gini score were chosen for the final model (“LDLPFC NVC,” “CEEV ratio,” and “*^w^* $\bar{D_{LDP}}$”). Model evaluation metrics on the single test set with 500 decision trees highlighted the model's validity. It achieved an accuracy of 85.71%, precision of 88.57%, recall of 85.71%, and an F1 score of 85.08%. For mean accuracy estimation, the model underwent 100 runs with different test‐train splits (80% train followed by 20% test) to ensure a comprehensive assessment of its performance. The aggregated metrics on the test set showed a mean accuracy of 68.0%, with an SD of 16.0%, indicating the reliability across diverse datasets, offering a more robust evaluation compared to a single test‐train split. The integration of LOOCV, random Forest, and multiple test‐train splits enhances the robustness of the model evaluation process, providing a comprehensive understanding of its performance on different datasets. The selected features and feature importance, along with the aggregated and individual metrics, collectively affirm the reliability and generalization capability of the developed classification model, determining new insights into the combined role of NVC, FC, and CEEVs in MCI etiopathology.

## 4 DISCUSSION

This study identified NVC dysfunction and decreased FC in participants with MCI compared to CN. These findings were localized to the LDLPFC. Exploratory study showed elevated ratio and concentration of CEEVs in MCI compared to CN. CEEVs significantly associated with increased WMH burden in participants with MCI. Following robust model and feature selection, NVC and FC in the LDLPFC, and CEEVs, were the most important variables in predicting MCI with high accuracy. These findings uncovered the alterations in cerebrovascular hemodynamics, and functional neuronal networks, while also showing CEEVs as a potential cerebrovascular pathologic marker that strongly associates with small vessel ischemic burden in participants with MCI.

We confirmed fluid cognitive dysfunction in our MCI cohort by identifying overall fluid cognitive deficits, impairment in working memory, inhibitory control and attention, cognitive flexibility, and processing speed measured by *n‐*back working memory test and NIH toolbox cognitive battery. The *n*‐back test has long‐standing validity as a working memory paradigm.^10^, ^38^ NIH toolbox cognitive battery has high discriminant ability that has found utility in clinical populations with cognitive disorders.^39^ Through these multitest cognitive domain evaluations, we provided strong certainty of group differences (MCI vs. CN) in fluid cognitive functioning and preserved crystalized abilities. Moreover, we showed that CEEVs were sensitive to fluid cognitive performance in our participants, indicating the potential dual‐role of CEEVs as biomarkers of cognitive performance and small vessel ischemic damage.

Reduced CBF from aging to MCI to dementia contributes to cognitive impairment pathophysiology^40^ and is one of the earliest events in dementia progression.^41^ However, limited and mixed evidence exists for changes in task‐activated hemodynamics between aging and MCI. Previous human studies showed no difference between MCI and CN in task‐evoked frontal cortex NVC responses.^42^, ^43^ Contrary to these data, others have reported decreased NVC in the frontal and prefrontal cortex.^44^, ^45^ Interventional trials to improve NVC and cognition are limited, albeit positive results that showed enhanced cognitive function and increased NVC responses after cocoa supplementation in participants with baseline impairment.^46^ The lack of intervention may be ascribed to the mixed results in human studies, highlighting the need for well‐designed, replicable work. Our data support NVC as a component of maintaining normal cognitive function, determining that participants with MCI have decreased NVC responses during working memory tasks compared to age‐matched controls. In line with our previous work, uncovering decreased NVC as a mechanism for aging‐associated cognitive decline compared to young adults,^10^ we show that NVC is further decreased in MCI compared to CN and is of critical importance in predicting cognitive impairment. Our findings from this cross‐sectional observational study provide increased translatability for clinical trials targeting the rescue of NVC responses to prevent progression from healthy aging to MCI.

Throughout the entire *n*‐back task, NVC dysfunction was localized to the LDLPFC in MCI compared to CN. These findings led us to determine the number, and strength of functional connections from the LDLPFC to the rest of the frontal cortex. The LDLPFC is an essential area for working memory performance and executive function regulation.^36^ In previous studies assessing FC of the PFC in participants with MCI, both increases^47^ and decreases were seen during cognitive tasks.^48^ Increases were interpreted as a compensatory action and decreases as a failure of the compensation mechanism. We report decreased task‐evoked global and LDLPFC number, and strength of functional connections in participants with MCI compared to CN, supporting a failure of compensation for an already impaired neurovasculature. In our recent work assessing NVC and FC changes in aging compared to young adults, we showed that in aging, task‐evoked NVC was decreased, and FC was increased, with a significant negative correlation.^10^ These data suggest that in healthy aging, increased coordinated activity between neuronal networks compensates for dysfunctional NVC. In MCI, NVC impairment is exacerbated, and FC is decreased, representing a loss of age‐related compensation mechanisms, resulting in cognitive impairment. This work allows us to postulate that both NVC and FC are essential in maintaining cognitive health in late life.

As disease modifying therapies are being developed for early and predementia stages, the ability to identify patients with MCI is of the utmost importance. We used NVC, FC, CEEV, and clinical data associated with MCI to determine an optimal classification model using machine learning (i.e., random Forest) integrated with LOOCV. In our robust, data‐driven modeling, we showed that LDLPFC NVC, CEEV ratio, and LDLPFC FC had the highest feature importance in MCI classification, even with age, sex, depression, and hypertension data incorporated in feature selection. Previous studies have shown machine learning predicting MCI diagnosis based on patient reported cognitive decline^49^ demographic and comorbidity variables,^50^ and resting state FC.^51^ Here, we determined that neurovascular health and FC are disrupted in MCI and that these features provide high discriminate value compared to demographic and comorbidity factors. Moreover, these findings place a great deal of importance for early identification of NVC disruption, as this disruption predates the loss of cognitive compensation mechanisms (i.e., FC)^10^ and MCI diagnosis. Prompt clinical trial opportunities targeting cerebrovascular health to rescue NVC, and potentially preserve FC^52^ compensatory measures, are essential for maintenance of cognitive health in our rapidly aging population.

Ratio of CEEVs from the total EEV population were higher in MCI compared to CN. CEEV isolation from patient plasma is a new and innovative technique that has only been assessed in multiple sclerosis (MS) patients^23^ prior to our study. The previous work from Mazzuco et al. showed increased plasma concentration of CEEVs in MS patients compared to healthy controls.^23^ Because the endothelium secretes more EVs when damaged or injured, our findings, in line with Mazzuco's previous work, reveal CEEV load as a potential marker of disease in CNS conditions. We also investigated the relationship between CEEVs, functional neuroimaging results, and WMH load. We determined that the ratio of CEEVs from the total EEV population has a significant and strong positive correlation with WMH burden, providing evidence of CEEV load as a potential marker of small vessel ischemic damage. In addition, current evidence indicates that the prevalence of EVs of larger sizes (>1000 nm) may be indicative of increased apoptotic mechanisms.^37^ EVs are classified into three categories based on size, exosomes (30‐150 nm), microvesicles (100‐1000 nm), and apoptotic bodies (1000‐5000 nm), and these subtypes differ in their biogenesis, release, intravesicular cargo, and function.^53^ However, criteria based on size overlaps between subtypes and is not exact in classifying EVs.^53^ Both exosomes and microvesicles are involved in cell‐to‐cell communication and diseased or dysfunctional cells package their cargo into these vesicles, which may have a role in dysfunction of nearby cellular systems.^53^ While we determined that concentration and ratio of CEEVs were increased in MCI compared to CN, there were no statistical differences in CEEV size ranges between the groups. Current findings in EV literature show exosome and microvesicle molecular cargo is associated with cognitive disease states,^54^, ^55^ potentially due to uptake from surrounding cells, compared to large, apoptotic bodies.^56^ This postulation is congruent with our distribution findings showing a trend of increased exosome and microvesicle distribution in MCI compared to CN.

Preclinical studies have identified that preserving the health of the cerebrovascular endothelium improves cognitive function.^57^ Here we show molecular and structural MRI correlations in MCI patients that may provide evidence of cerebrovascular endothelial dysfunction. Future directions to elucidate MCI molecular alterations is to assess the cargo (i.e., protein, miRNA) within CEEVs. Recent evidence has revealed that in early‐onset MCI patients, exosome protein cargo correlated with cerebral spinal fluid tau levels, WMHs, brain atrophy and cognition scores.^58^, ^59^ Addressing alterations in molecular cargo of CEEVs will uncover pathways for how increased concentration of CEEVs may cause cerebrovascular damage, and provide insight into new, mechanistically targeted therapy.[Fig alz14072-fig-0007]

Cross‐sectional human physiological studies always pose a challenge in balancing groups with respect to comorbidities. While groups were not matched for comorbidities, no significant differences between groups were seen. Comorbidities that had higher prevalence in MCI compared to CN, such as hypertension and depression, were added to the MCI classification model and showed low feature importance. An additional challenge in human studies is balancing the groups for demographic factors, particularly those that influence brain structure and function. While all participants were not both age‐ and sex‐matched, 15 of the 20 age‐matched controls were also sex matched to participants with MCI. We showed no significant difference in education levels in Fisher's exact test when splitting groups between bachelor's degree and higher versus less than a bachelor's degree of educational attainment. However, there were more higher educational achievers in the CN group compared to MCI, but not significantly different. Along with our findings of FC, we suggest the assessment of structural connectivity by diffusion tensor imaging in future studies. This will aid in unraveling the subcortical white matter neurodegenerative processes that associate with functional alterations in cognitive impairment. In this study, we determined intricate shifts in neurovascular brain function, as well as a potentially novel biomarker that needs further investigation of protein and miRNA cargo for pathway analysis, and differentiation of cargo based on CEEV size. Future work should also investigate pan‐specific markers for CEEVs to increase accuracy of cell‐of‐origin identification. Our results should be considered a building block for expanding mechanistic insights of CEEVs and for development of interventional trials.

Overall, our study highlights that disruption of NVC and FC are prominent factors in MCI pathophysiology. Additionally, we determined the relevance of CEEVs to ischemic burden in participants with MCI. Potential therapeutic directions are warranted from our findings to ultimately improve cerebrovascular function to rescue NVC and FC, thereby preserving cognitive function in high‐risk individuals. Our findings of increased levels of CEEVs provide promise for future mechanistic studies and pathway analysis of cerebrovascular endothelial dysfunction. Additionally, CEEV analysis may have the potential to provide diagnostic and prognostic markers of cerebrovascular disease and neurocognitive disorders, warranting future, longitudinal studies.

## CONFLICT OF INTEREST STATEMENT

The authors declare no conflicts of interest. Author disclosures are available in the supporting information.

## CONSENT STATEMENT

All measurements and protocols were approved by the Institutional Review Board of the University of Oklahoma Health Sciences Center (#14585). Written informed consent was received prior to participation. This study was performed in accordance with the ethical standards of the Declaration of Helsinki.

## Supporting information

Supporting Information

Supporting Information

## References

[1] Nichols E , Steinmetz JD , Vollset SE , et al. Estimation of the global prevalence of dementia in 2019 and forecasted prevalence in 2050: an analysis for the Global Burden of Disease Study 2019. Lancet Public Health. 2022;7:e105‐e125. doi:10.1016/S2468-2667(21)00249-8 34998485 PMC8810394

[2] Mattson MP , Arumugam TV . Hallmarks of brain aging: adaptive and pathological modification by metabolic states. Cell Metab. 2018;27:1176‐1199. doi:10.1016/j.cmet.2018.05.011 29874566 PMC6039826

[3] Perneczky R . Dementia treatment versus prevention. Dialogues Clin Neurosci. 2019;21:43‐51. 10.31887/DCNS.2019.21.1/rperneczky 31607779 PMC6780357

[4] Bai W , Chen P , Cai H , et al. Worldwide prevalence of mild cognitive impairment among community dwellers aged 50 years and older: a meta‐analysis and systematic review of epidemiology studies. Age Ageing. 2022;51. doi:10.1093/ageing/afac173 35977150

[5] Knopman DS , Amieva H , Petersen RC , et al. Alzheimer disease. Nat Rev Dis Primers . 2021;7:33. doi:10.1038/s41572-021-00269-y 33986301 PMC8574196

[6] Reinvang I , Grambaite R , Espeseth T . Executive dysfunction in MCI: subtype or early symptom. J Alzheimer's Dis. 2012;2012:936272. doi:10.1155/2012/936272 PMC3369514 22693679

[7] Campbell NL , Unverzagt F , LaMantia MA , Khan BA , Boustani MA . Risk factors for the progression of mild cognitive impairment to dementia. Clin Geriatr Med. 2013;29:873‐893. doi:10.1016/j.cger.2013.07.009 24094301 PMC5915285

[8] Zhu WM , Neuhaus A , Beard DJ , Sutherland BA , DeLuca GC . Neurovascular coupling mechanisms in health and neurovascular uncoupling in Alzheimer's disease. Brain. 2022;145:2276‐2292. doi:10.1093/brain/awac174 35551356 PMC9337814

[9] Tarantini S , Tran CHT , Gordon GR , Ungvari Z , Csiszar A . Impaired neurovascular coupling in aging and Alzheimer's disease: contribution of astrocyte dysfunction and endothelial impairment to cognitive decline. Exp Gerontol. 2017;94:52‐58. doi:10.1016/j.exger.2016.11.004 27845201 PMC5429210

[10] Mukli P , Pinto CB , Owens CD , et al. Impaired neurovascular coupling and increased functional connectivity in the frontal cortex predict age‐related cognitive dysfunction. Adv Sci (Weinh). 2023:e2303516. doi:10.1002/advs.202303516 38155460 PMC10962492

[11] Kisler K , Nelson AR , Montagne A , Zlokovic BV . Cerebral blood flow regulation and neurovascular dysfunction in Alzheimer disease. Nat Rev Neurosci. 2017;18:419‐434. doi:10.1038/nrn.2017.48 28515434 PMC5759779

[12] Friston KJ , Kahan J , Biswal B , Razi A . A DCM for resting state fMRI. Neuroimage. 2014;94:396‐407. doi:10.1016/j.neuroimage.2013.12.009 24345387 PMC4073651

[13] Farràs‐Permanyer L , Guàrdia‐Olmos J , Peró‐Cebollero M . Mild cognitive impairment and fMRI studies of brain functional connectivity: the state of the art. Front Psychol. 2015;6:1095. doi:10.3389/fpsyg.2015.01095 26300802 PMC4523742

[14] Lin L , Xing G , Han Y . Advances in resting state neuroimaging of mild cognitive impairment. Front Psychiatry. 2018;9:671. doi:10.3389/fpsyt.2018.00671 30574100 PMC6291484

[15] Wolters FJ , Zonneveld HI , Hofman A , et al. Cerebral perfusion and the risk of dementia. Circulation. 2017;136:719‐728. doi:10.1161/CIRCULATIONAHA.117.027448 28588075

[16] Tang T , Huang L , Zhang Y , Li Z , Liang S . Aberrant pattern of regional cerebral blood flow in mild cognitive impairment: a meta‐analysis of arterial spin labeling magnetic resonance imaging. Front Aging Neurosci. 2022;14. doi:10.3389/fnagi.2022.961344 PMC9475306 36118708

[17] Calcetas AT , Thomas KR , Edmonds EC , et al. Increased regional white matter hyperintensity volume in objectively‐defined subtle cognitive decline and mild cognitive impairment. Neurobiol Aging. 2022;118:1‐8. doi:10.1016/j.neurobiolaging.2022.06.002 35809348 PMC9838569

[18] Li Q , Yang Y , Reis C , et al. Cerebral small vessel disease. Cell Transplant. 2018;27:1711‐1722. doi:10.1177/0963689718795148 30251566 PMC6300773

[19] Huang H , Zhao K , Zhu W , Li H , Zhu W . Abnormal cerebral blood flow and functional connectivity strength in subjects with white matter hyperintensities. Front Neurol. 2021;12:1‐13. doi:10.3389/fneur.2021.752762 PMC8564178 34744987

[20] Lee Y , EL Andaloussi S , Wood MJA . Exosomes and microvesicles: extracellular vesicles for genetic information transfer and gene therapy. Hum Mol Genet. 2012;21:R125‐R134. doi:10.1093/hmg/dds317 22872698

[21] Jansen F , Nickenig G , Werner N . Extracellular vesicles in cardiovascular disease. Circ Res. 2017;120:1649‐1657. doi:10.1161/CIRCRESAHA.117.310752 28495995

[22] Viswanathan A , Rocca WA , Tzourio C . Vascular risk factors and dementia: how to move forward? Neurology. 2009;72:368‐374. doi:10.1212/01.wnl.0000341271.90478.8e 19171835 PMC2677504

[23] Mazzucco M , Mannheim W , Shetty SV , Linden JR . CNS endothelial derived extracellular vesicles are biomarkers of active disease in multiple sclerosis. Fluids Barriers CNS. 2022;19:13. doi:10.1186/s12987-021-00299-4 35135557 PMC8822708

[24] Visconte C , Golia MT , Fenoglio C , et al. Plasma microglial‐derived extracellular vesicles are increased in frail patients with Mild Cognitive Impairment and exert a neurotoxic effect. Geroscience. 2023;45:1557‐1571. doi:10.1007/s11357-023-00746-0 36725819 PMC10400496

[25] Zhao Z , Nelson AR , Betsholtz C , Zlokovic BV . Establishment and dysfunction of the blood‐brain barrier. Cell. 2015;163:1064‐1078. 26590417 10.1016/j.cell.2015.10.067 PMC4655822

[26] Kynast J , Lampe L , Luck T , et al. White matter hyperintensities associated with small vessel disease impair social cognition beside attention and memory. J Cereb Blood Flow Metab. 2018;38:996‐1009. doi:10.1177/0271678x17719380 28685621 PMC5999004

[27] Owens CD , Bonin Pinto C , Mukli P , et al. Vascular mechanisms leading to progression of mild cognitive impairment to dementia after COVID‐19: protocol and methodology of a prospective longitudinal observational study. PLOS ONE. 2023;18:e0289508. doi:10.1371/journal.pone.0289508 37535668 PMC10399897

[28] Haatveit BC , Sundet K , Hugdahl K , Ueland T , Melle I , Andreassen OA . The validity of d prime as a working memory index: results from the “Bergen n‐back task”. J Clin Exp Neuropsychol. 2010;32:871‐880. doi:10.1080/13803391003596421 20383801

[29] Santosa H , Zhai X , Fishburn F , Huppert T . The NIRS Brain AnalyzIR Toolbox. Algorithms. 2018;11:73. 38957522 10.3390/a11050073 PMC11218834

[30] Mukli P , Csipo T , Lipecz A , et al. Sleep deprivation alters task‐related changes in functional connectivity of the frontal cortex: a near‐infrared spectroscopy study. Brain Behav. 2021;11:e02135. doi:10.1002/brb3.2135 34156165 PMC8413792

[31] Kocsis L , Herman P , Eke A . The modified Beer‐Lambert law revisited. Phys Med Biol. 2006;51:N91‐N98. doi:10.1088/0031-9155/51/5/n02 16481677

[32] Racz FS , Mukli P , Nagy Z , Eke A . Increased prefrontal cortex connectivity during cognitive challenge assessed by fNIRS imaging. Biomed Opt Express. 2017;8:3842‐3855. doi:10.1364/boe.8.003842 28856054 PMC5560845

[33] Cheng Y , Zeng Q , Han Q , Xia W . Effect of pH, temperature and freezing‐thawing on quantity changes and cellular uptake of exosomes. Protein Cell. 2018;10:295‐299. doi:10.1007/s13238-018-0529-4 PMC6418301 29616487

[34] Guo Y , Pai M , Xue B , Lu W . Bidirectional association between depressive symptoms and mild cognitive impairment over 20 years: evidence from the Health and Retirement Study in the United States. J Affect Disord. 2023;338:449‐458. doi:10.1016/j.jad.2023.06.046 37356735

[35] Harrington KD , Dang C , Lim YY , et al. The effect of preclinical Alzheimer's disease on age‐related changes in intelligence in cognitively normal older adults. Intelligence. 2018;70:22‐29. doi:10.1016/j.intell.2018.07.004

[36] Barbey AK , Koenigs M , Grafman J . Dorsolateral prefrontal contributions to human working memory. Cortex. 2013;49:1195‐1205. doi:10.1016/j.cortex.2012.05.022 22789779 PMC3495093

[37] Caruso S , Poon IKH . Apoptotic cell‐derived extracellular vesicles: more than just debris. Front Immunol. 2018;9:1486. doi:10.3389/fimmu.2018.01486 30002658 PMC6031707

[38] Jaeggi SM , Buschkuehl M , Perrig WJ , Meier B . The concurrent validity of the N‐back task as a working memory measure. Memory. 2010;18:394‐412. doi:10.1080/09658211003702171 20408039

[39] Hackett K , Krikorian R , Giovannetti T , et al. Utility of the NIH Toolbox for assessment of prodromal Alzheimer's disease and dementia. Alzheimers Dement (Amst). 2018;10:764‐772. doi:10.1016/j.dadm.2018.10.002 30505926 PMC6247399

[40] Zhang H , Wang Y , Lyu D , et al. Cerebral blood flow in mild cognitive impairment and Alzheimer's disease: a systematic review and meta‐analysis. Ageing Res Rev. 2021;71:101450. doi:10.1016/j.arr.2021.101450 34419673

[41] Korte N , Nortley R , Attwell D . Cerebral blood flow decrease as an early pathological mechanism in Alzheimer's disease. Acta Neuropathol. 2020;140:793‐810. doi:10.1007/s00401-020-02215-w 32865691 PMC7666276

[42] Nakamura S , Yomota S , Ito H , et al. A novel cognitive function scale using functional near‐infrared spectroscopy for evaluating cognitive dysfunction. J Alzheimers Dis. 2021;81:1579‐1588. doi:10.3233/jad-210072 33967049 PMC8293658

[43] Li R , Rui G , Chen W , Li S , Schulz PE , Zhang Y . Early detection of Alzheimer's disease using non‐invasive near‐infrared spectroscopy. Front Aging Neurosci. 2018;10:366. doi:10.3389/fnagi.2018.00366 30473662 PMC6237862

[44] Niu HJ , Li X , Chen YJ , Ma C , Zhang JY , Zhang ZJ . Reduced frontal activation during a working memory task in mild cognitive impairment: a non‐invasive near‐infrared spectroscopy study. CNS Neurosci Ther. 2013;19:125‐131. doi:10.1111/cns.12046 23279823 PMC6493442

[45] Yeung MK , Sze SL , Woo J , et al. Reduced frontal activations at high working memory load in mild cognitive impairment: near‐infrared spectroscopy. Dement Geriatr Cogn Disord. 2016;42:278‐296. doi:10.1159/000450993 27784013

[46] Sorond FA , Hurwitz S , Salat DH , Greve DN , Fisher ND . Neurovascular coupling, cerebral white matter integrity, and response to cocoa in older people. Neurology. 2013;81:904‐909. doi:10.1212/WNL.0b013e3182a351aa 23925758 PMC3885215

[47] Yu JW , Lim SH , Kim B , et al. Prefrontal functional connectivity analysis of cognitive decline for early diagnosis of mild cognitive impairment: a functional near‐infrared spectroscopy study. Biomed Opt Express. 2020;11:1725‐1741. doi:10.1364/boe.382197 32341843 PMC7173911

[48] Nguyen T , Kim M , Gwak J , et al. Investigation of brain functional connectivity in patients with mild cognitive impairment: a functional near‐infrared spectroscopy (fNIRS) study. J Biophotonics. 2019;12:e201800298. doi:10.1002/jbio.201800298 30963713

[49] Gómez‐Ramírez J , Ávila‐Villanueva M , Fernández‐Blázquez MÁ . Selecting the most important self‐assessed features for predicting conversion to mild cognitive impairment with random forest and permutation‐based methods. Sci Rep. 2020;10:20630. doi:10.1038/s41598-020-77296-4 33244011 PMC7692490

[50] Zhong X , Yu J , Jiang F , et al. A risk prediction model based on machine learning for early cognitive impairment in hypertension: development and validation study. Front Public Health. 2023;11:1143019. doi:10.3389/fpubh.2023.1143019 36969637 PMC10034177

[51] Khazaee A , Ebrahimzadeh A , Babajani‐Feremi A . Application of advanced machine learning methods on resting‐state fMRI network for identification of mild cognitive impairment and Alzheimer's disease. Brain Imaging Behav. 2016;10:799‐817. doi:10.1007/s11682-015-9448-7 26363784

[52] Vipin A , Loke YM , Liu S , et al. Cerebrovascular disease influences functional and structural network connectivity in patients with amnestic mild cognitive impairment and Alzheimer's disease. Alzheimers Res Ther. 2018;10:82. doi:10.1186/s13195-018-0413-8 30121086 PMC6098837

[53] Doyle LM , Wang MZ . Overview of extracellular vesicles, their origin, composition, purpose, and methods for exosome isolation and analysis. Cells. 2019;8:1‐24. doi:10.3390/cells8070727 PMC6678302 31311206

[54] Pulliam L , Sun B , Mustapic M , Chawla S , Kapogiannis D . Plasma neuronal exosomes serve as biomarkers of cognitive impairment in HIV infection and Alzheimer's disease. J Neurovirol. 2019;25:702‐709. doi:10.1007/s13365-018-0695-4 30610738 PMC7372698

[55] McKeever PM , Schneider R , Taghdiri F , et al. MicroRNA Expression levels are altered in the cerebrospinal fluid of patients with young‐onset Alzheimer's disease. Mol Neurobiol. 2018;55:8826‐8841. doi:10.1007/s12035-018-1032-x 29603092 PMC6208843

[56] Liu YJ , Wang C . A review of the regulatory mechanisms of extracellular vesicles‐mediated intercellular communication. Cell Commun Signal. 2023;21:77. doi:10.1186/s12964-023-01103-6 37055761 PMC10100201

[57] Tarantini S , Valcarcel‐Ares MN , Toth P , et al. Nicotinamide mononucleotide (NMN) supplementation rescues cerebromicrovascular endothelial function and neurovascular coupling responses and improves cognitive function in aged mice. Redox Biol. 2019;24:101192. doi:10.1016/j.redox.2019.101192 31015147 PMC6477631

[58] Cano A , Esteban‐de‐Antonio E , Bernuz M , et al. Plasma extracellular vesicles reveal early molecular differences in amyloid positive patients with early‐onset mild cognitive impairment. J Nanobiotechnology. 2023;21:54. doi:10.1186/s12951-023-01793-7 36788617 PMC9930227

[59] Sharygin D , Koniaris LG , Wells C , Zimmers TA , Hamidi T . Role of CD14 in human disease. Immunology. 2023;169:260‐270. doi:10.1111/imm.13634 36840585 PMC10591340

