# Supplementary material for: Neurovascular coupling, functional connectivity, and cerebrovascular endothelial extracellular vesicles as biomarkers of mild cognitive impairment
Source: Alzheimers Dement. 2024 Jul 3;20(8):5590–606. doi: 10.1002/alz.14072 (PMC11350141; doi:10.1002/alz.14072)
Supplement: Supplementary file 1 — Supporting Information [file ALZ-20-5590-s001.docx]

**Supplementary material for:**

**Neurovascular coupling, functional connectivity and cerebrovascular endothelial extracellular vesicles as biomarkers of mild cognitive impairment**

Cameron D. Owens,^1,2,#^ Camila Bonin Pinto,^1,2,#^ Peter Mukli,^1,2,3,4,#^ Rafal Gulej,^1,2,#^ Faddi Saleh Velez,^5^ Sam Detwiler,^1,2^ Lauren Olay,^1,2^ Jordan R. Hoffmeister,^6^ Zsofia Szarvas,^1,2,4^ Mihaly Muranyi,^1,2,4^ Anna Peterfi,^1,2,4^ Ana Clara da C. Pinaffi-Langley,^1,7^ Cheryl Adams,^1,2^ Jason Sharps,^5^ Zalan Kaposzta,^1,2,4^ Calin I. Prodan,^5,8^ Angelia C. Kirkpatrick,^8,9^ Stefano Tarantini,^1,2,4,10,11^ Anna Csiszar,^1,2,4^ Zoltan Ungvari,^1,2,4,10^ Ann L. Olson,^12^ Guangpu Li,^12^ Priya Balasubramanian,^1,2,11^ Veronica Galvan,^1,12^ Andrew Bauer,^2^ Zachary A. Smith,^2^ Tarun W. Dasari,^9^ Shawn Whitehead,^13^ Manoj R. Medapti,^13^ Fanny M. Elahi,^14,15^ Aikaterini Thanou,^16,17^ Andriy Yabluchanskiy*^1,2,10,11^

**Affiliations:**

^1^Oklahoma Center for Geroscience and Healthy Brain Aging, University of Oklahoma Health Sciences Center, Oklahoma City, OK, United States of America

^2^Vascular Cognitive Impairment and Neurodegeneration Program, Department of Neurosurgery, University of Oklahoma Health Sciences Center, Oklahoma City, OK, United States of America

^3^Department of Physiology, Faculty of Medicine, Semmelweis University, Budapest, Hungary

^4^International Training Program in Geroscience, Doctoral School of Basic and Translational Medicine/Department of Public Health, Semmelweis University, Budapest, Hungary

^5^Department of Neurology, University of Oklahoma Health Sciences Center, Oklahoma City, OK, United States of America

^6^Neuropsychology Service, Department of Psychiatry and Behavioral Sciences, University of Oklahoma Health Sciences Center, Oklahoma City, OK, United States of America

^7^Department of Nutritional Sciences, College of Allied Health, University of Oklahoma Health Sciences Center, Oklahoma City, OK, United States of America

^8^Veterans Affairs Medical Center, Oklahoma City, OK, United States of America

^9^Cardiovascular Disease Section, Department of Medicine, University of Oklahoma Health Sciences Center, Oklahoma City, OK, United States of America

^10^Department of Health Promotion Sciences, College of Public Health, University of Oklahoma Health Sciences Center, Oklahoma City, OK, United States of America

^11^Peggy and Charles Stephenson Cancer Center, University of Oklahoma Health Sciences Center, Oklahoma City, OK, United States of America

^12^Department of Biochemistry and Molecular Biology, University of Oklahoma Health Sciences Center, Oklahoma City, OK, United States of America

^13^Vulnerable Brain Laboratory, Department of Anatomy & Cell Biology, Schulich School of Medicine & Dentistry, London, OH, Canada

^14^Icahn School of Medicine at Mount Sinai, New York, NY, United States of America

^15^James J. Peters Department of Veterans Affairs Medical Center, Bronx, NY, United States of America

^16^Oklahoma Medical Research Foundation, Oklahoma City, OK, United States of America

^17^Department of Medicine, University of Oklahoma Health Sciences Center, Oklahoma City, OK, United States of America

* Correspondence to:

Andriy Yabluchanskiy, MD, PhD

1122 NE 13^th^ St.

University of Oklahoma Health Sciences Center,

Oklahoma City, OK, 73117, United States of America

[Andriy-Yabluchanskiy@ouhsc.edu](mailto:Andriy-Yabluchanskiy@ouhsc.edu) – 405-271-8001

^#^ denotes co-first authorship

**Declarations of interest:** none

**Supplementary Figures**

**
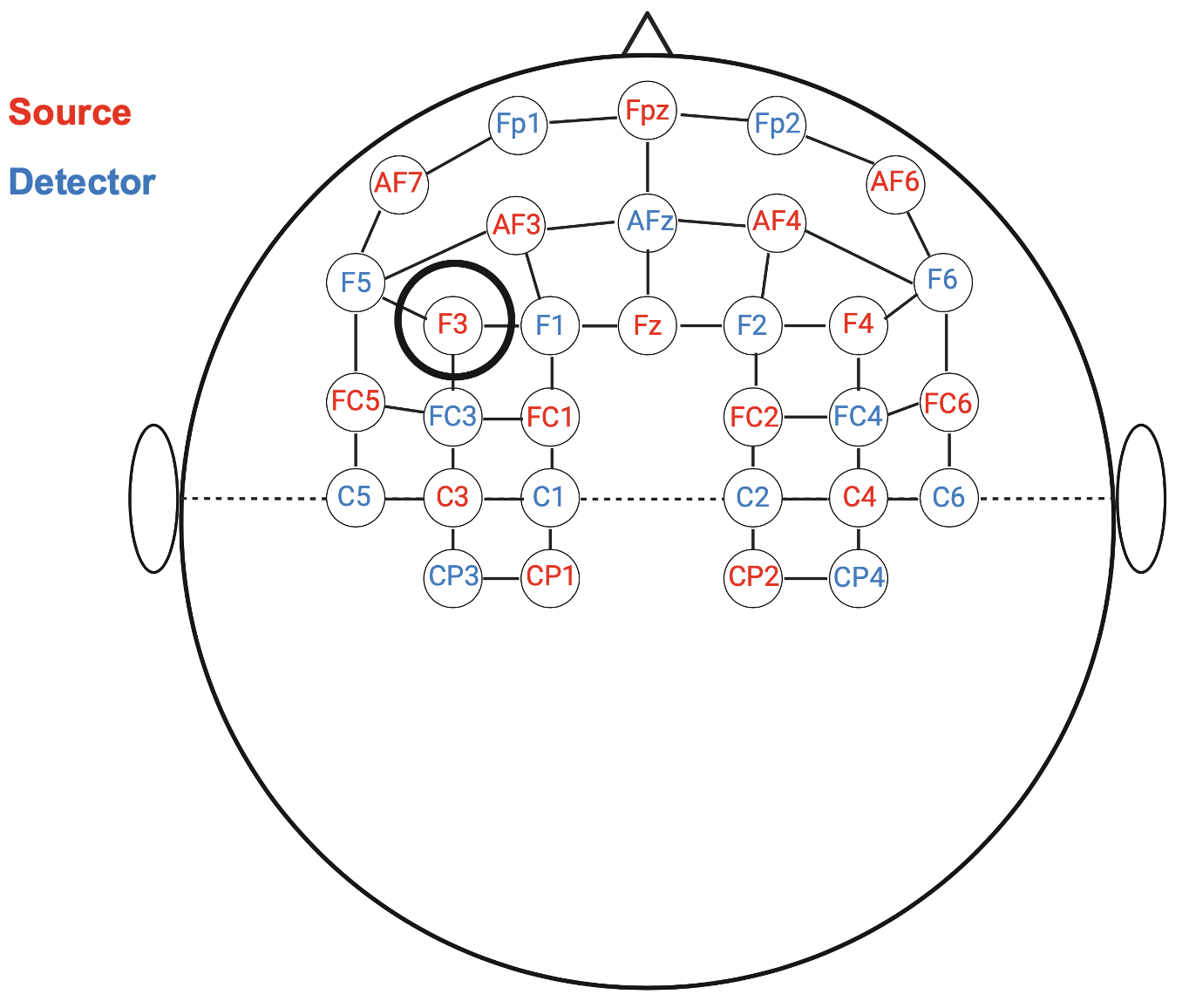
**

**Supplementary figure 1. fNIRS montage.** Topographical fNIRS set up used to measure NVC responses and FC were defined by standard optodes placement from 48 channels. Source-detector pairs have 3cm of separation and are in accordance with the international 10-20 system. All channels cover the prefrontal and frontal cortex. The black circle surrounding F3 represents the LDLPFC, the region of interest for the current study.

**
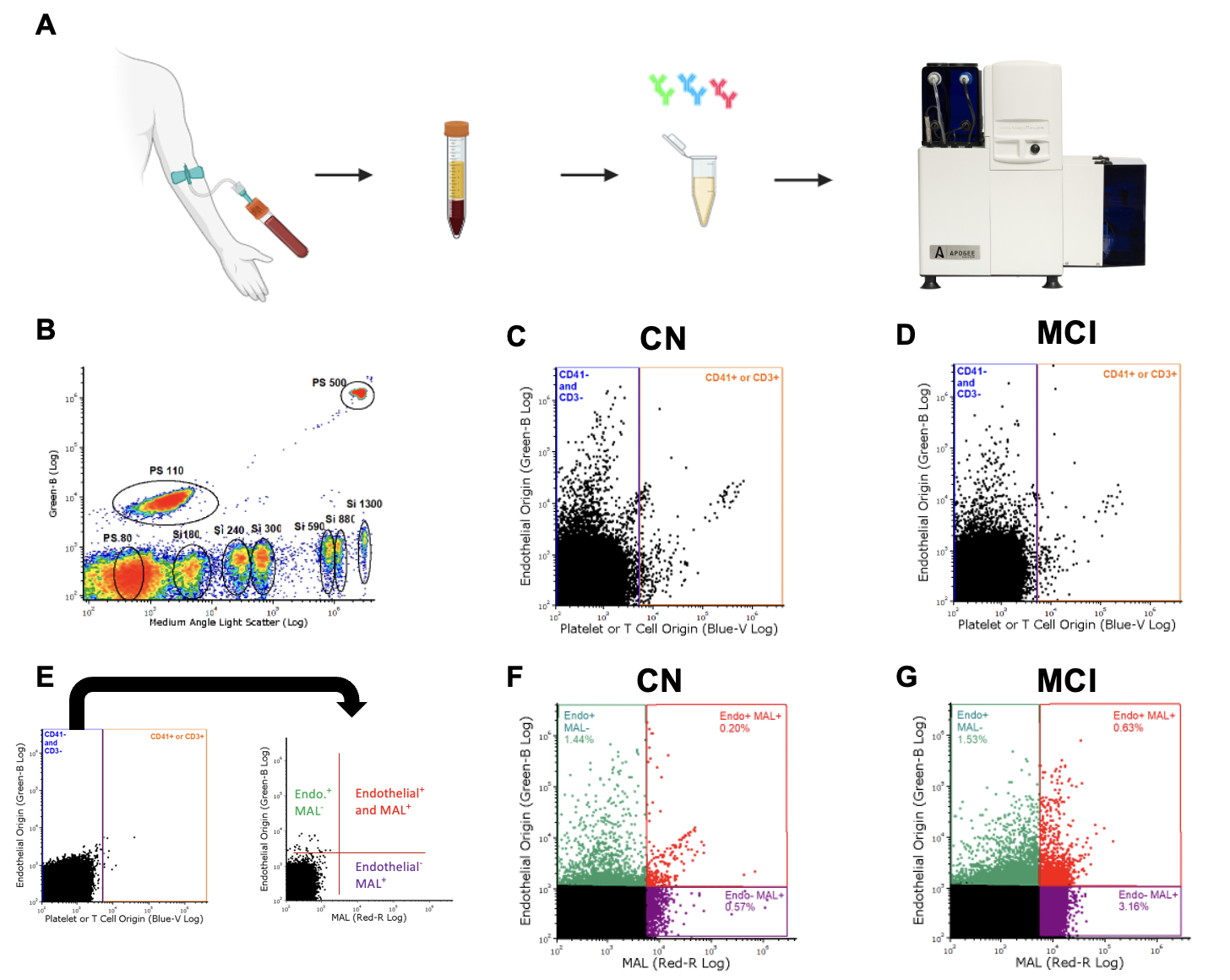
**

**Supplementary figure 2. Blood draw, processing, extracellular vesicle measurement and analysis.** (**A**) Blood is collected from patients, processed according to our protocol (see main text), and aliquoted to 100 µl until EV measurement. For EV preparation, 10 µl of plasma were added to 80 µl of 0.22 µl filtered phosphate saline (PBS) and stained with 10 µl of antibody master mix (endothelial, platelet and lymphocyte origin). Following sample mixing for two hours on ice, concentration and ratio of EVs were measured using ApogeeFlow Micro-Plus flow cytometer. (**B**) silica and polystyrene beads in size from 80 to 1300 nm. (**C-E**) Representative images of gating out plasma and lymphocyte origin. (**F, G**) Representative images of CD31+/ CD105+/CD144+ and MAL+ extracellular vesicles following gating out CD41 and CD3.

**
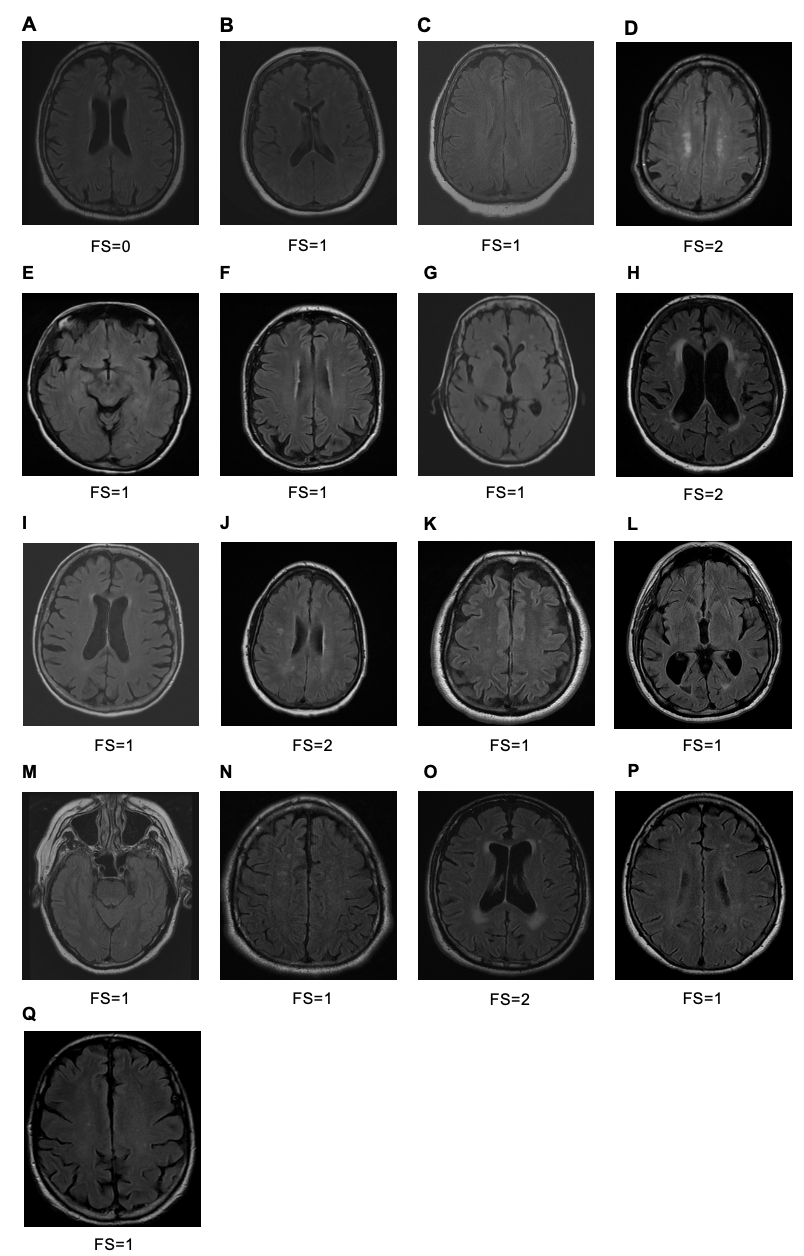
**

**Supplementary figure 3. Participants with MCI subcortical ischemic lesion grading. (A-Q)** MCI participant Fazekas scale grading for deep white matter hyperintensities. Each panel represents a representative image of a patient with MCI.

**
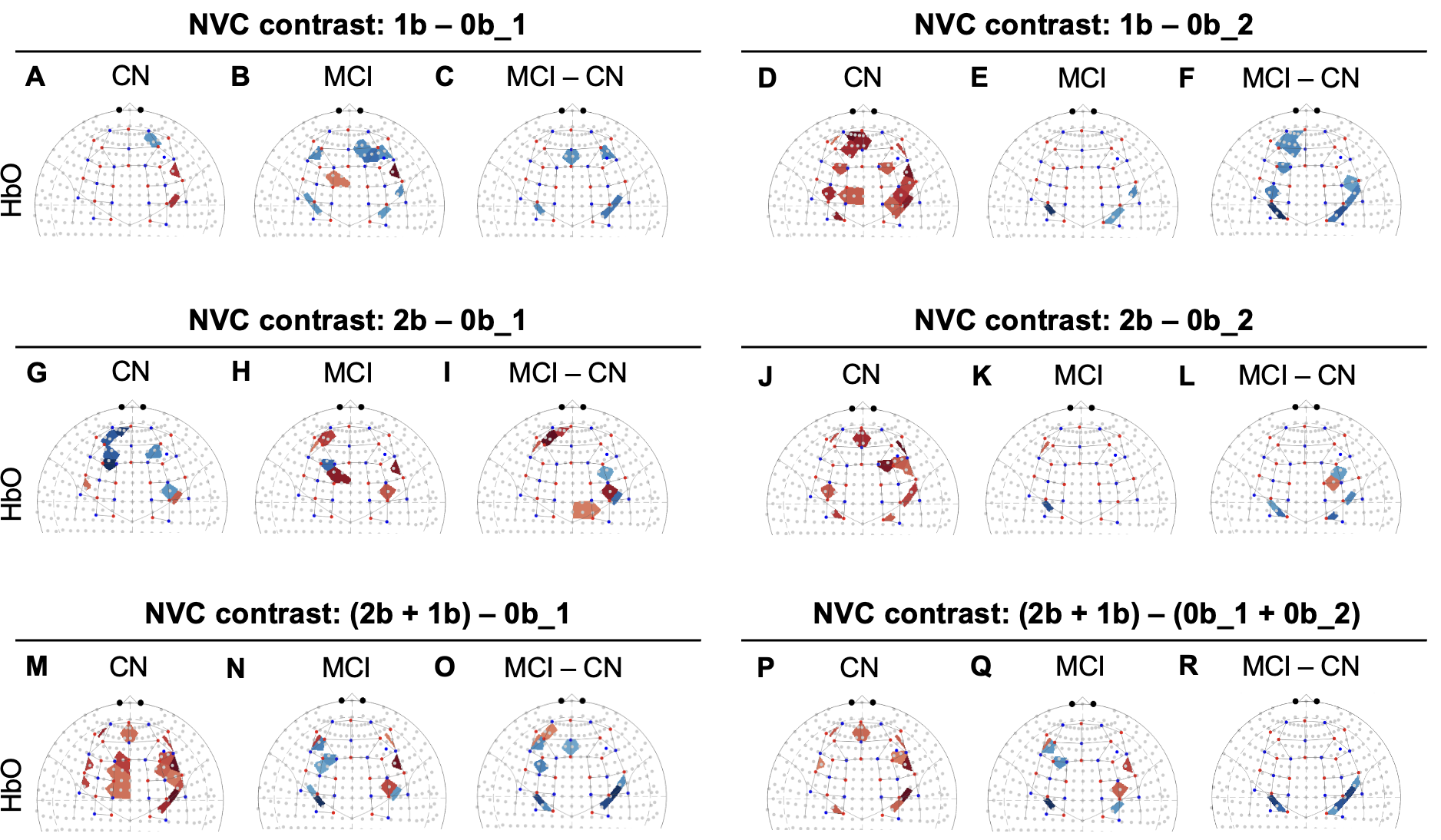
**

**Supplementary figure 4. Effect of n-back condition on NVC.** Panels **A-B** show the statistical contrast between 1-back (1b) and first 0-back (0b_1) for age-matched controls (CN) and patients with MCI. Group comparison **(C)** show decreased NVC in medial and right prefrontal cortex (PFC) in MCI compared to CN. Panels **D-E** show the statistical contrast between 1b and second 0-back (0b_2) for CN and patients with MCI. Group comparison **(F)** show decreased NVC in medial and left PFC and LDLPFC in MCI compared to CN. Panels **G-H** show the statistical contrast between 2-back (2b) and 0b_1 for CN and patients with MCI. Group comparison **(I)** show increased left PFC NVC and decreased right-dorsolateral prefrontal cortex (RDLPFC) NVC in MCI compared to CN. Panels **J-K** show the statistical contrast between 2b and 0b_2 for CN and patients with MCI. Group comparison **(L)** shows decreased RDLPFC NVC in MCI compared to CN. Panels **M-N** show the statistical contrast between (2b + 1b) – 0b_1 for CN and patients with MCI to determine the effects of the most cognitively challenging tasks relative to the first, least challenging task. Group comparison **(O)** shows decreased NVC in LDLPFC and medial PFC in MCI compared to CN. Panels **P-Q** show the statistical contrast between (2b + 1b) – (0b_1 + 0b_2) for CN and patients with MCI to determine the effects of the most cognitively challenging tasks relative to the least challenging tasks. Group comparison **(R)** shows no change in PFC NVC response in MCI compared to CN. t-contrast maps were generated using Brain AnalyzIR toolbox implemented pipeline based on General Linear Model approach. For further details see main text. *t*-values for contrast maps had a cut off value at *q*<0.05 (obtained after false discovery rate correction).

**
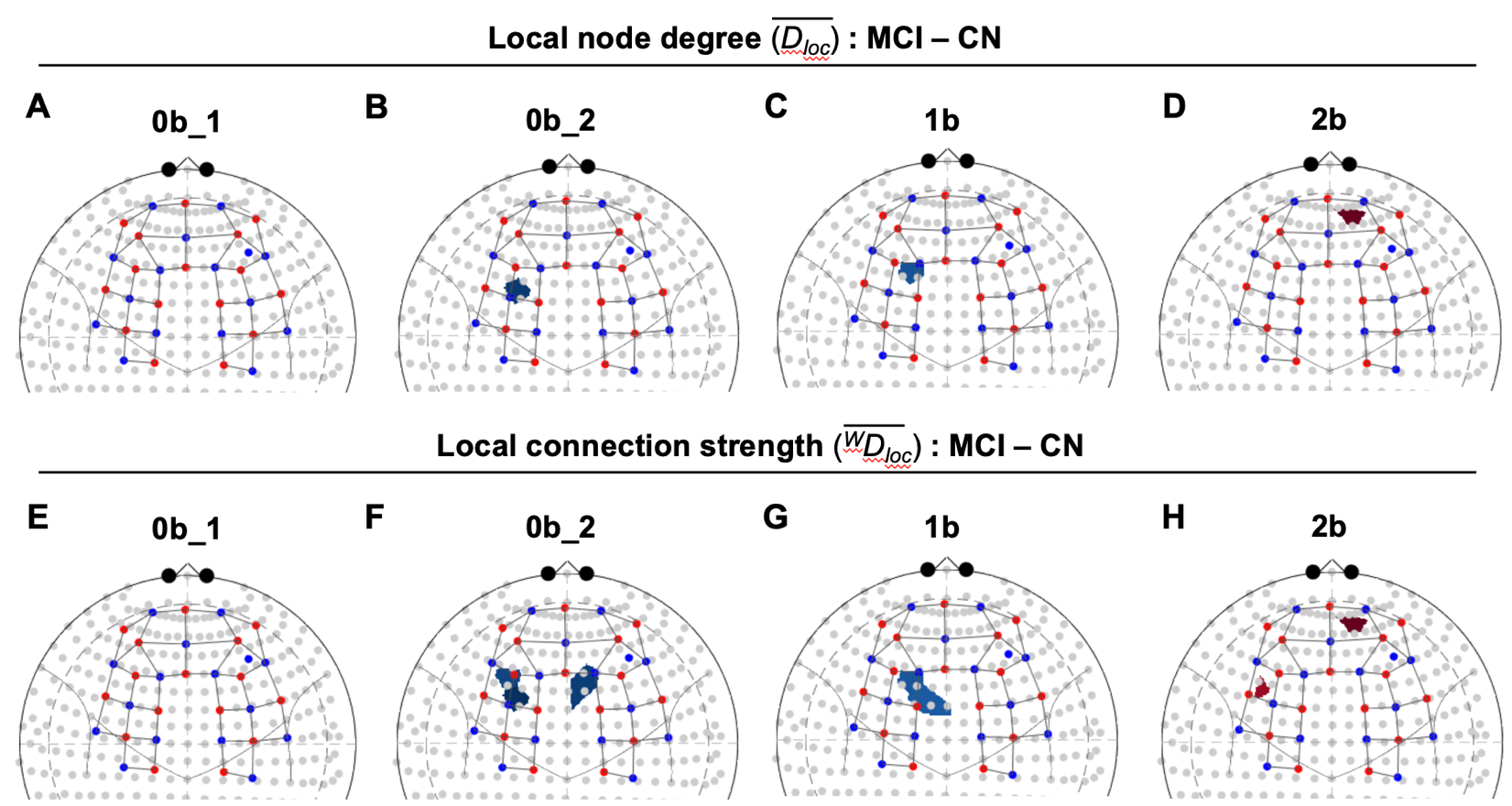
**

**Supplementary figure 5. Local functional connections and connection strength are decreased in MCI.** (**A-D**) Normalized local node degree and (**E-H**) local functional strength measures of surrogate thresholded (p<0.05) connections were calculated for each group (CN and MCI), fNIRS channel, and averaged for each condition (0b_1, 0b_2, 1b, 2b). Significance thresholded (*p*<0.05) *t-*contrasts were plotted on to frontal cortex maps for statistical comparisons: (**A, E**) MCI 0b_1 – CN 0b_1, (**B, F**) MCI 0b_2 – CN 0b_2, (**C, G**) MCI 1b – CN 1b, (**D, H**) MCI 2b – CN 2b. These data determined that there were decreases (blue shaded areas) in LDLPFC functional connections and connection strength during 0b_2 and 1b task and increased medial PFC functional connectivity during 2b in MCI compared to CN (*p*<0.05).

**
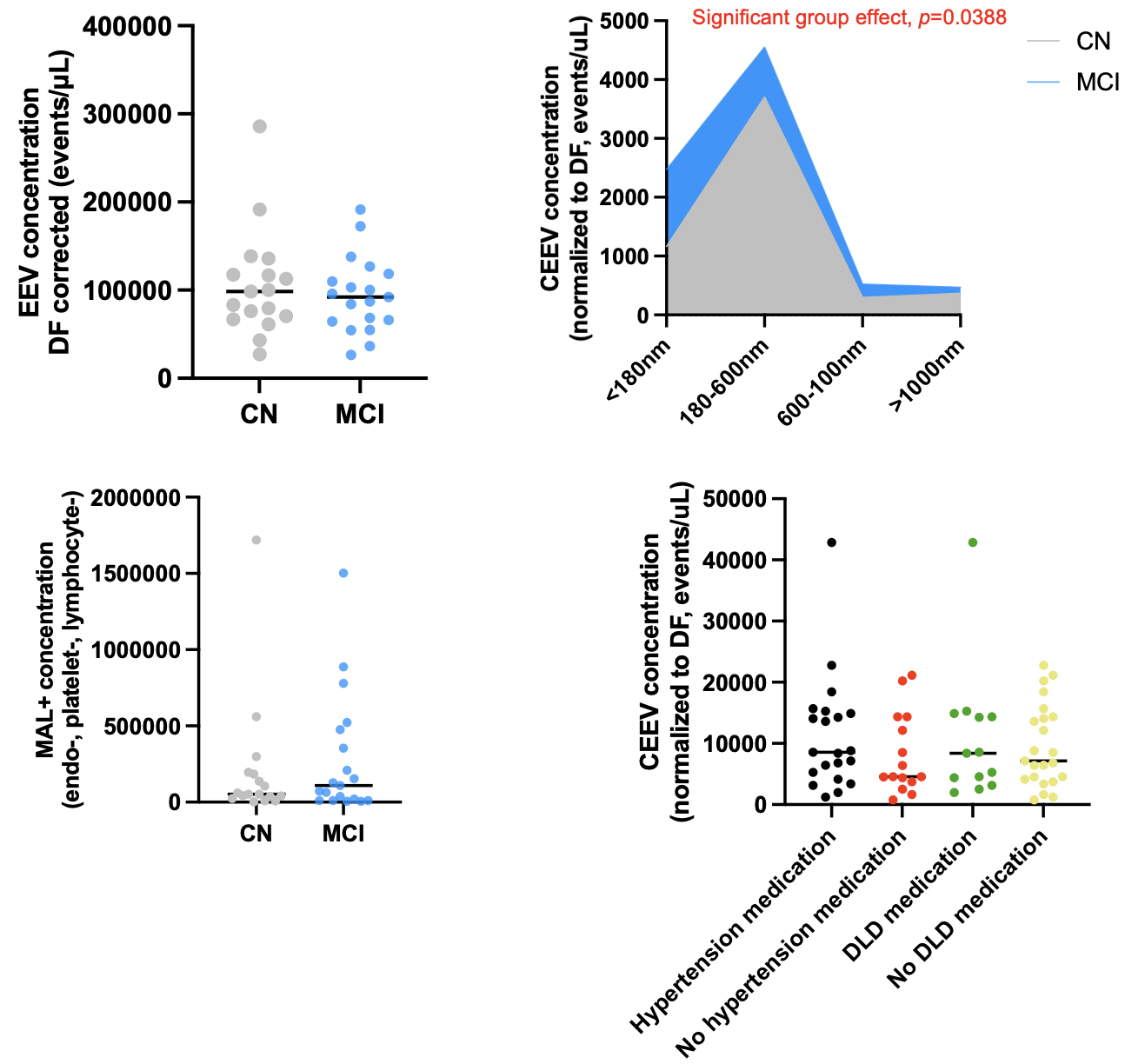
**

**Supplementary Figure 6. EEV concentration, CEEV size distribution, and MAL+/endo-/platelet-/lymphocyte- concentration. (A)** Concentration of total dilution factor (DF) corrected EEV concentration in MCI compared to CN. **(B)** Average size distribution at specified ranges predetermined from silica and polystyrene bead sizes. **(C)** Dilution factor corrected MAL+ concentration in patient plasma after gating out endothelial, platelet and lymphocyte markers.

**
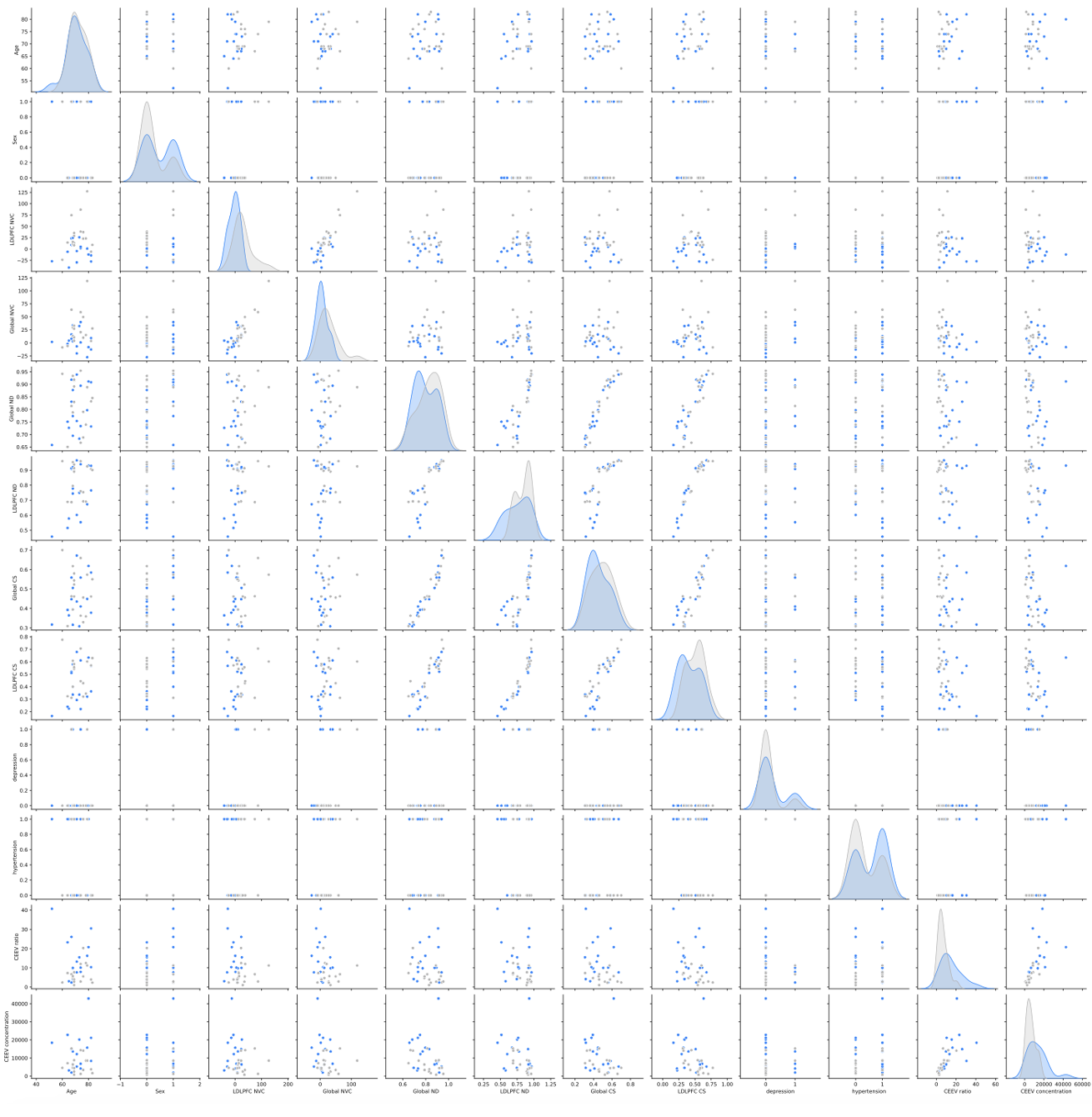
**

**Supplementary figure 7. Patients with MCI have distinctive distributions of final model features compared to CN.** The matrix shows all variables used in feature selection process to identify the most informative variables. Additionally, distribution of participant data is separated by group, blue (MCI) and gray (CN), for each variable (e.g., Age) and relationships between variables (e.g., Age x Sex). Most informative features in the final model (LDLPFC NVC, CEEV ratio, and LDLPFC binary node degree) indicate a group distinctive distribution.

**Supplementary table 1. MCI etiology**

|  | | | | | | | | | |
| --- | --- | --- | --- | --- | --- | --- | --- | --- | --- |
|  | | **Other** | | **Cerebrovascular disease** | | **Alzheimer's disease** | | **N/A** | |
| MCI (*n*) |  | 8.00 |  | 5.00 |  | 5.00 |  | 2.00 |  |
|  | | | | | | | | | |
| Note. Medical neurology records from patients indicated that patients were of ‘Unknown etiology/other’, MCI due to cerebrovascular disease, MCI due to Alzheimer’s disease, and two participants did not have record listed presumed etiology. | | | | | | | | | |
|  | | | | | | | | | |

**Supplementary table 2. Functional neuroimaging and CEEV associations**

| Correlation Matrix | | | | | | | | | | | | | | | |
| --- | --- | --- | --- | --- | --- | --- | --- | --- | --- | --- | --- | --- | --- | --- | --- |
|  | |  | | **LDLPFC NVC** | | **LDLPFC ND** | | **LDLPFC CS** | | **CEEV ratio** | | **CEEV concentration** | | **Fluid cognition** | |
| LDLPFC NVC |  | Spearman's rho |  | — |  |  |  |  |  |  |  |  |  |  |  |
|  |  | df |  | — |  |  |  |  |  |  |  |  |  |  |  |
|  |  | p-value |  | — |  |  |  |  |  |  |  |  |  |  |  |
| LDLPFC ND |  | Spearman's rho |  | 0.066 |  | — |  |  |  |  |  |  |  |  |  |
|  |  | df |  | 32 |  | — |  |  |  |  |  |  |  |  |  |
|  |  | p-value |  | 0.710 |  | — |  |  |  |  |  |  |  |  |  |
| LDLPFC CS |  | Spearman's rho |  | 0.092 |  | 0.959 | *** | — |  |  |  |  |  |  |  |
|  |  | df |  | 32 |  | 32 |  | — |  |  |  |  |  |  |  |
|  |  | p-value |  | 0.604 |  | < .001 |  | — |  |  |  |  |  |  |  |
| CEEV ratio |  | Spearman's rho |  | -0.157 |  | -0.295 |  | -0.368 | * | — |  |  |  |  |  |
|  |  | df |  | 32 |  | 32 |  | 32 |  | — |  |  |  |  |  |
|  |  | p-value |  | 0.374 |  | 0.091 |  | 0.033 |  | — |  |  |  |  |  |
| CEEV concentration |  | Spearman's rho |  | -0.119 |  | -0.348 | * | -0.396 | * | 0.854 | *** | — |  |  |  |
|  |  | df |  | 32 |  | 32 |  | 32 |  | 32 |  | — |  |  |  |
|  |  | p-value |  | 0.500 |  | 0.044 |  | 0.021 |  | < .001 |  | — |  |  |  |
| Fluid cognition |  | Spearman's rho |  | 0.413 | * | 0.277 |  | 0.339 | * | -0.528 | ** | -0.502 | ** | — |  |
|  |  | df |  | 32 |  | 32 |  | 32 |  | 32 |  | 32 |  | — |  |
|  |  | p-value |  | 0.015 |  | 0.113 |  | 0.050 |  | 0.001 |  | 0.002 |  | — |  |
| Note. * p < .05, ** p < .01, *** p < .001 | | | | | | | | | | | | | | | |
|  | | | | | | | | | | | | | | | |

Abbreviations: CEEV ratio: cerebrovascular extracellular vesicle percentage of total endothelial extracellular vesicles; ND LDLPFC: binary node degree of left dorsolateral prefrontal cortex; CS LDLPFC: weighted node degree of left dorsolateral prefrontal cortex; NVC LDLPFC: neurovascular coupling response from the LDLPFC.
